# Organotransition Metal Chemistry of Terpenes: Syntheses, Structures, Reactivity and Molecular Rearrangements

**DOI:** 10.3390/molecules29061409

**Published:** 2024-03-21

**Authors:** Michael J. McGlinchey

**Affiliations:** School of Chemistry, University College Dublin, Belfield, D04 V1W8 Dublin, Ireland; michael.mcglinchey@ucd.ie

**Keywords:** nickel and iron carbonyls, allyl-palladium couplings, pheromones, ring-strained terpenes, McMurry coupling, epoxidations, guaiazulene, X-ray crystallography

## Abstract

The impact of organometallic chemistry on the terpene field only really blossomed in the 1960s and 1970s with the realisation that carbon–carbon bond formation under mild conditions could be achieved by using nickel or iron carbonyls as synthetic reagents. Concomitantly, the development of palladium derivatives capable of the controlled coupling of isoprene units attracted the attention of numerous highly talented researchers, including future Nobel laureates. We discuss briefly how early work on the syntheses of simple monoterpenes soon progressed to sesquiterpenes and diterpenes of increasing complexity, such as humulene, flexibilene, vitamin A, or pheromones of commercial value, in particular those used in perfumery (muscone, lavandulol), or grandisol and red scale pheromone as replacements for harmful pesticides. As the field progressed, there has been more emphasis on developing organometallic routes to enantiopure rather than racemic products, as well as gaining precise mechanistic data on the transformations, notably the course of metal-promoted molecular rearrangements that have long been a feature of terpene chemistry. We note the impact of the enormously enhanced analytical techniques, high-field NMR spectroscopy and X-ray crystallography, and their use to re-examine the originally proposed structures of terpenes and their organometallic derivatives. Finally, we highlight the very recent ground-breaking use of the crystalline sponge method to acquire structural data on low-melting or volatile terpenes. The literature cited herein covers the period 1959 to 2023.

## 1. Introduction

Terpenoids (or isoprenoids) found commonly in essential oils of plants have long fascinated chemists, not only for their outstanding structural diversity, but also for the versatility of their molecular rearrangements. The elucidation of their structures and biosynthetic pathways, and the rationalisation of their rearrangement behaviour is one of the most satisfying successes of organic chemistry and owes much to such pioneers as Otto Wallach, Leopold Ružička, Konrad Bloch and Feodor Lynen, and John Cornforth (Nobel laureates in 1910, 1939, 1964 and 1975, respectively). While the pleasantly agreeable odours of many volatile terpenes have led to their widespread use in the perfume industry, in their own natural world, they can function as protection against predators, as pheromones for sexual attraction, as trail pheromones, and in a multitude of other ways [1]. We note that, strictly speaking, *terpenes* are oligomers or polymers of isoprene (C_5_H_8_), and depending on the number of isoprene units are categorized as monoterpenes (C_10_H_16_), sesquiterpenes (C_15_H_24_), diterpenes (C_20_H_32_), etc. Those molecules with a different formula, such as geraniol (C_10_H_18_O) or citral (C_10_H_16_O), are known as monoterpenoids rather than monoterpenes [2].

**Scheme 1:**
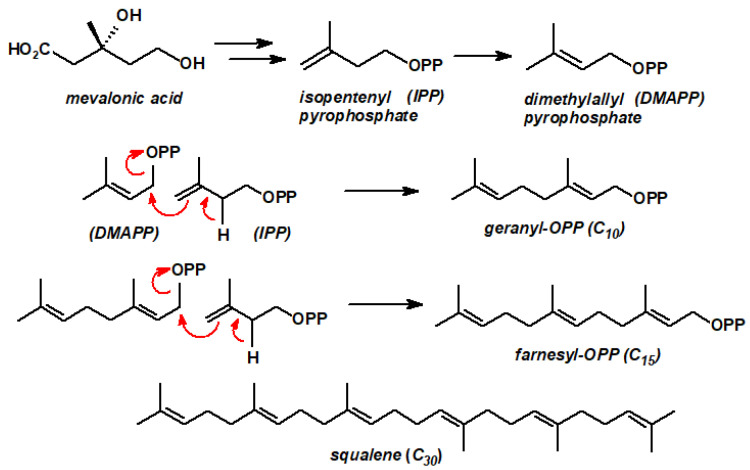
Biosynthetic route to monoterpenes and sesquiterpenes.

Although terpenes appear superficially to be assembled simply by combining isoprenes, in fact, the crucial intermediate in their biosynthetic pathway is *mevalonic acid* (derived originally from acetate) that is converted in several steps into isopentenyl pyrophosphate (IPP), which itself undergoes an enzyme-catalysed rearrangement to form its more reactive dimethylallyl pyrophosphate isomer (DMAPP). Nucleophilic attack by IPP at the C-1 site of DMAPP with the concomitant elimination of pyrophosphate, an excellent leaving group, yields geranyl-OPP, the precursor to myriad (C_10_) monoterpenes. As shown in Scheme 1, subsequent alkylation of geranyl-OPP by IPP leads to farnesyl-OPP, the precursor of the sesquiterpenes (C_15_), while the head-to-head coupling of farnesyl units produces squalene (C_30_), a progenitor of the steroid skeleton.

**Scheme 2:**
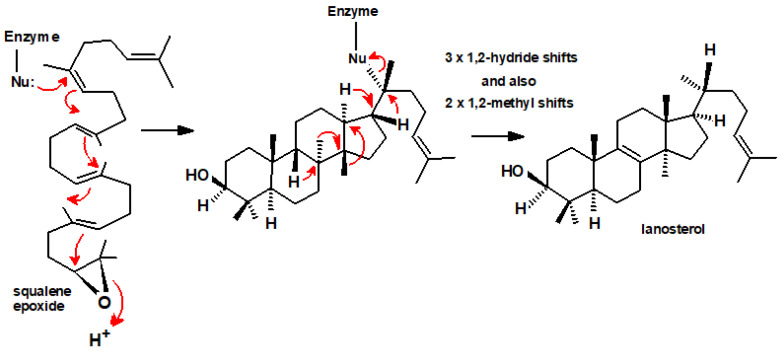
Rearrangement of 2,3-squalene epoxide to form lanosterol.

As shown in Scheme 2, the 2,3-epoxide of the triterpene *squalene*, when folded appropriately on an enzyme surface, undergoes a series of hydride and methyl 1,2-shifts leading to lanosterol (Scheme 2), and ultimately to human sex hormones, such as testosterone and estradiol [3].

**Figure 1:**
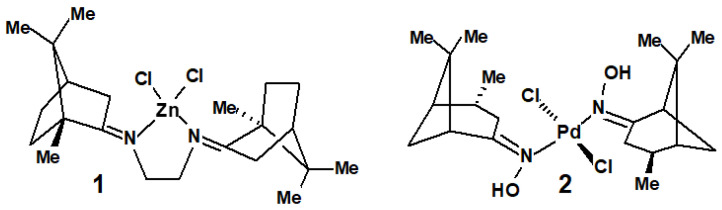
Examples of coordination compounds containing a terpenoid substituent.

Since these biosynthetic routes to terpenes are mediated by enzymes, they yield enantiopure products that find extensive applications in catalytic asymmetric syntheses. This aspect of their chemistry, i.e., the formation of coordination compounds possessing chiral terpenoid ligands, such as those based on the camphor or pinane framework, **1** or **2**, respectively (Figure 1), has been comprehensively reviewed [4].

However, our focus here is on the organometallic chemistry of terpenoids, with the aim of providing a short introduction to the field, and an overview of its development in recent decades. We begin with the pioneering work on the syntheses of simple terpenoids in the 1960s and 1970s, and the subsequent extension to more complex molecular targets. In all cases, the organometallic steps in the synthetic sequences are those highlighted. We then discuss the reactions and molecular rearrangements of terpenoids when treated with organometallic reagents, finally moving towards the current status and future goals of the field.

## 2. Synthetic Overview

**Scheme 3:**
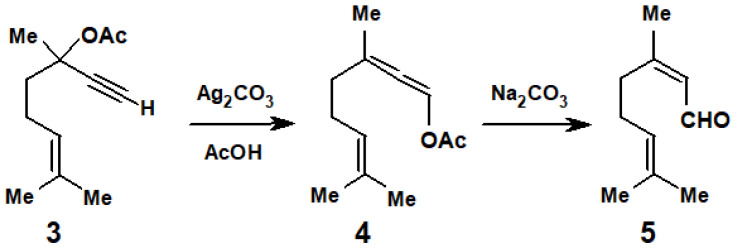
Synthesis of citral mediated by silver carbonate.

The use of transition metals in terpenoid synthesis is a long-established approach. Typically, in 1959, a large-scale route to *citral* was developed in which the α-acetoxy-alkyne, **3**, was treated with silver carbonate in acetic acid to form the allene **4**, which yielded citral, **5**, upon saponification (Scheme 3) [5].

This area gained considerable impetus in the 1960s and 1970s when the development of new and improved transition-metal-based carbon–carbon coupling reactions, many of them catalytic in nature, was a major focus of a number of leading investigators, several of whom received Nobel Prizes for their seminal contributions. In many instances, researchers chose terpenoids as synthetic targets to illustrate the utility and versatility of their own new-found reaction sequences.

One general method takes advantage of the ability of metal carbonyls, such as Fe(CO)_5_ or Ni(CO)_4_, to discard CO ligands and undergo oxidative addition reactions with carbon–halogen linkages, while another major approach uses palladium(II) salts as precursors to Pd(0) systems, generally stabilized by phosphines; another, now very widely adopted, procedure is based on the generation of π-allyl-nickel or -palladium intermediates. It is, noteworthy, however, that many researchers now focus their synthetic efforts on the use of first-row (3d) transition elements [6] rather than heavy precious-metal catalysts, not only for their lower cost and ready availability, but also for their low toxicity. We shall describe representative examples of all the abovementioned methodologies where they have been used in synthetic routes to isoprenoids.

## 3. Organonickel Complexes as Coupling Reagents

### 3.1. Dehalogenations Using Ni(CO)_4_

**Scheme 4:**
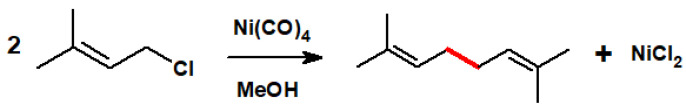
First reported coupling of allylic halides by nickel tetracarbonyl.

The earliest use of Ni(CO)_4_ in carbon–carbon bond formation is to be found in a Belgian Patent filed in 1943 [7]; but the first journal publication appeared in 1951, when Webb and Borcherdt reported that a wide range of allylic halides could be coupled to form the corresponding diallyl (1,5-diene) products with the elimination of carbon monoxide (Scheme 4) [8]. However, they emphasized the need for great care in handling such a volatile (b.p. 43 °C) and toxic reagent.

**Scheme 5:**
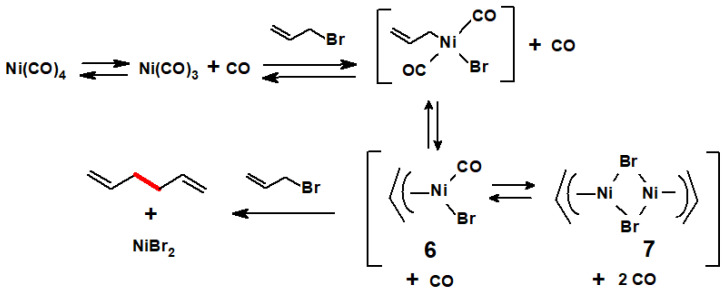
Intermediates in the coupling of allyl halides with nickel tetracarbonyl.

This reaction requires the loss of CO ligands to generate vacant sites available for oxidative addition by the allyl halide, and the formation of an (η^3^-allyl)nickel carbonyl halide, **6**, in equilibrium with the diallyl complex, **7**, both of which react irreversibly with allyl bromide in a polar solvent to ultimately yield the 1,5-diene product and NiBr_2_ (Scheme 5).

**Scheme 6:**
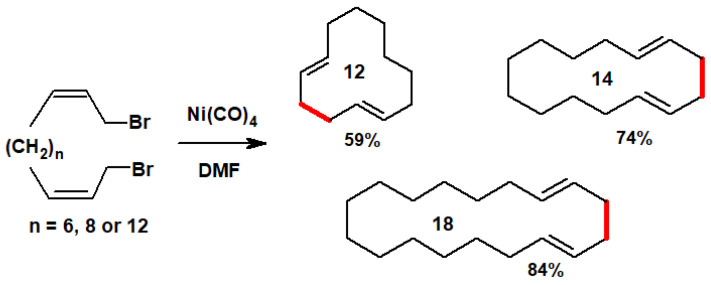
Formation of large-ring 1,5-dienes using Ni(CO)_4_ as a coupling reagent.

Despite the potentially hazardous nature of Ni(CO)_4_, this concept was brilliantly exploited [9,10] by E.J. Corey (Nobel 1990), who extended it to long-chain α,ω-dibromo-diene systems, thereby forming large-ring 1,5-dienes containing up to 18 carbons in modest-to-good yields [11], as in Scheme 6.

**Scheme 7:**
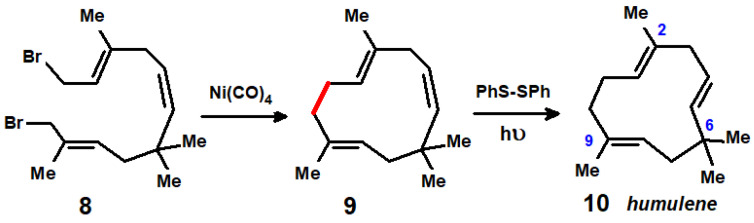
Final stages in the synthesis of humulene by Corey and Hamanaka.

This approach offered a route to *humulene*, (2,6,6,9-tetramethyl-*trans*,*trans*,*trans*-cycloundeca-1,4,8-triene), a monocyclic sesquiterpene containing an 11-membered ring and found in the essential oils of *Humulus lupulus* (hops), from which it derives its name. To this end, treatment of the dibromide **8** with Ni(CO)_4_ in N-methylpyrrolidone delivered **9**, possessing a *cis* C4-C5 double bond; subsequent photolysis with diphenyl disulphide in cyclohexane brought about the required *cis*-*trans* isomerisation to form humulene, **10**, in modest yield (Scheme 7) [12]. A second report, from the group of O.P Vig in Chandighar, India, using essentially this same route, appeared in 1976 [13].

**Scheme 8:**
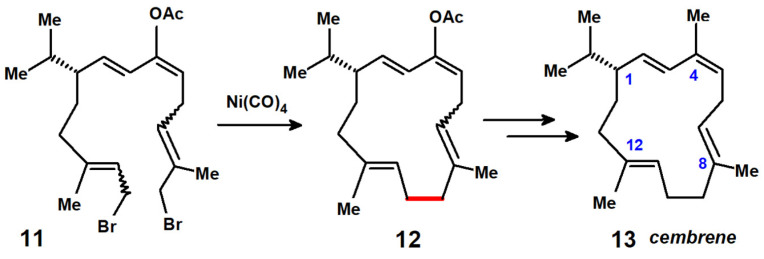
Ring-closing sequence in Dauben’s route to cembrene.

**Figure 2:**
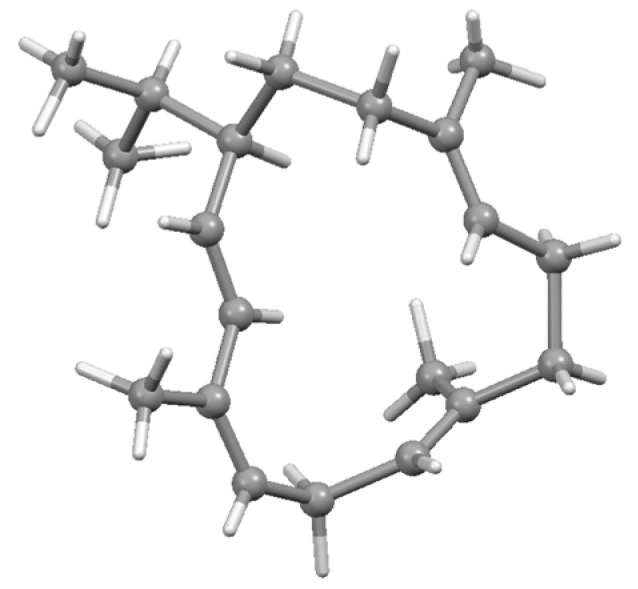
Molecular structure of cembrene.

In related work, Dauben’s group used this approach to prepare *cembrene* (1-isopropyl-4,8,12-trimethylcyclododeca-2,4,7,11-tetraene), a monocyclic diterpene found in pine oil and which contains a 14-membered ring [14]. The key feature shown in Scheme 8 depicts the nickel tetracarbonyl-mediated ring closure of the acetate derivative **11** to form **12**. To complete the synthesis, Jones oxidation of the acetate functionality to ketone, reaction with MeLi and the dehydration of the resulting alcohol delivered cembrene, **13**, spectroscopically identical to the authentic material whose X-ray crystal structure appears as Figure 2 [15].

### 3.2. π-Allyl-Nickel Complexes

**Scheme 9:**
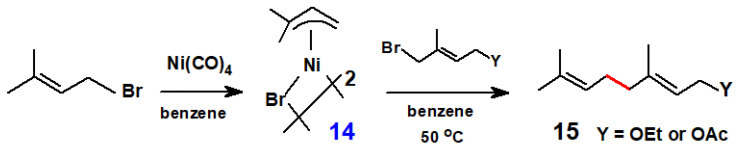
Synthesis of geranyl acetate from the allyl-nickel bromide dimer, **14**.

Continuing in this vein, Sato reported that the treatment of (1,1-dimethyl-π-allyl)nickel bromide dimer, **14**, with 4-acetoxy-1-bromo-2-methyl-2-butene (or its 4-ethoxy analogue) gave geranyl acetate (or geranyl ethyl ether), **15**, in moderate yield (Scheme 9) [16].

**Scheme 10:**
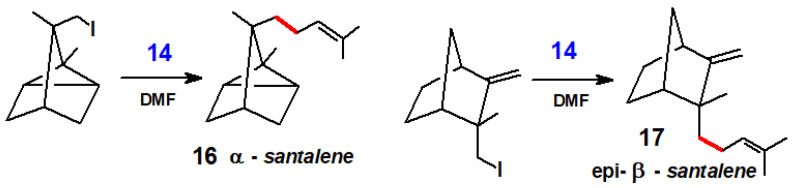
Final steps in the syntheses of α- and epi-β-santalenes using the allyl-nickel bromide dimer, **14**.

Likewise, Corey and Semmelhack used this approach to complete the syntheses of *α-santalene*, **16**, and *epi-β-santalene*, **17**, by prenylation of their iodomethyl side chains (Scheme 10) [10].

The direct involvement of π-allyl-nickel intermediates in such processes was unequivocally demonstrated in now classic studies by the Wilke group at the Max Planck-Institut für Kohlenforschung in Mülheim, Germany. They not only isolated and characterized many such species derived from 1,3-butadiene and a source of “naked nickel” (from a Ni(0) precursor, e.g., bis(1,5-cyclooctadiene)nickel), but also elucidated how the various coupling products are dependent on the associated ligands (phosphines, phosphites, CO) and on the ligand-to-metal ratio. This was eloquently reviewed in a virtuoso contribution by Günther Wilke himself [17].

### 3.3. Syntheses of Grandisol and of Muscone

**Scheme 11:**
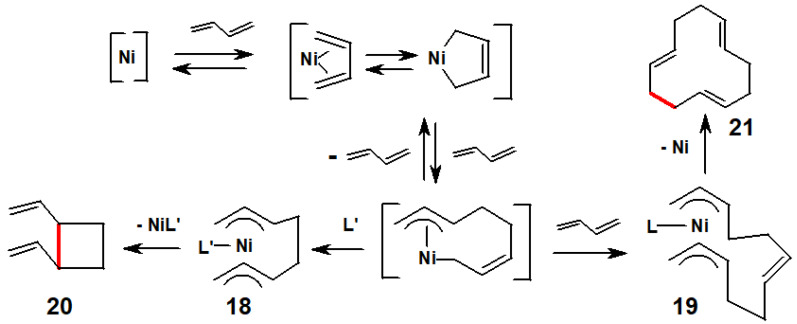
Nickel-promoted dimerisation and trimerisation of 1,3-butadiene.

For our specific purposes, we concentrate on two such π-allyl-nickel complexes: (i) the C_8_H_12_Ni system, **18**, derived from the coupling of two butadiene units, and (ii) the C_12_H_18_Ni homologue, **19**, arising from incorporation of three butadiene monomers. In the former case, elimination of the nickel moiety yields *cis*-1,2-divinylcyclobutane, **20**, whereas the latter leads to 1,5,9-cyclododecatriene, **21** (Scheme 11).

**Scheme 12:**
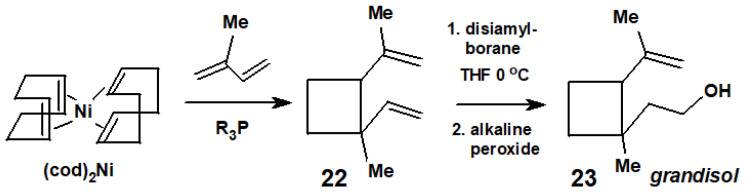
Billups’s synthesis of (±)-grandisol.

Since the widespread use of pesticides has been severely criticised because of its harmful effects on human and wildlife, an alternative approach to pest control has been proposed, involving the use of insect pheromones. To this end, the formation of *cis*-1,2-divinylcyclobutane from **18** prompted Ed Billups from Rice University in Houston, Texas, to carry out the same reaction, but starting from isoprene, with the goal of preparing the important monoterpenoid *grandisol*, a key constituent of the male boll weevil pheromone. Gratifyingly, the reaction of a large excess of isoprene with (cod)_2_Ni and tris(2-biphenylyl) phosphite furnished the desired dimer, **22**, in a 15% yield. Subsequent hydroboration with disiamylborane in THF at 0 °C followed by treatment of the organoborane with alkaline peroxide yielded (±)-grandisol, **23** (52%), as shown in Scheme 12 [18].

**Scheme 13:**
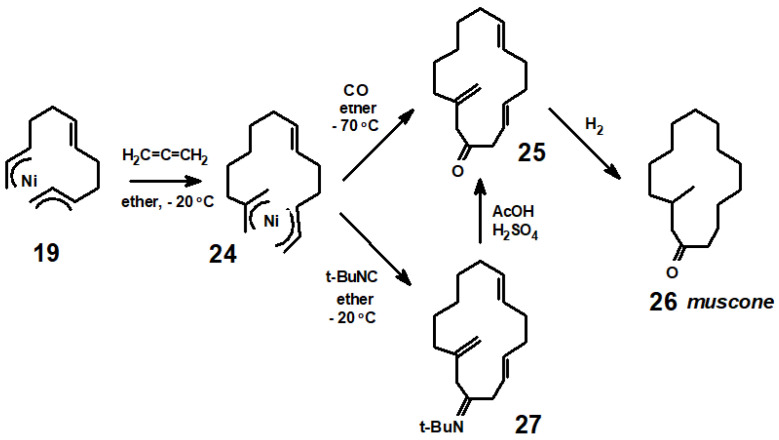
Synthesis of (±)-muscone from dodecatrienylnickel.

The synthetic availability of dodecatrienylnickel, **19**, and its known ring closure to 1,5,9-cyclododecatriene, **21**, offered the possibility of an incorporation of an additional small unit, such as allene, thereby extending the molecular skeleton to form a 14-membered carbon chain. As shown in Scheme 13, this was successfully achieved and led to the extended bis-allyl-nickel complex, **24** [19]. Subsequent incorporation of carbon monoxide resulted in the formation of the 15-membered ring species, **25**, that also possessed a ketone functionality, and the hydrogenation of the double bonds yielded (±)*-muscone*, **26**, that is obtainable in small quantities as its (*R*)-(-)-enantiomer from a glandular secretion of the male musk deer.

In an improved synthesis of muscone, also by the Baker/Cookson group [20], the incorporation of allene formed **24**; subsequently, the reaction with *tert*-butyl isocyanide formed the imine, **27**, that was then converted into the previously prepared ketone, **25**, thereby raising the overall yield to 40%. Muscone is now manufactured commercially, starting from (+)-citronellal, and is widely used in perfumery.

### 3.4. Organocobalt Complexes as Substitutes for Ni(CO)_4_

**Scheme 14:**
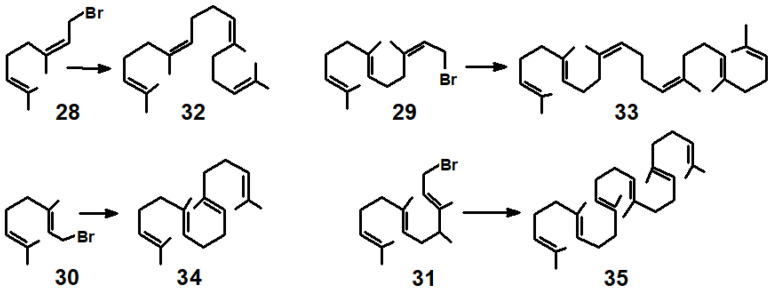
Coupling of geranyl and farnesyl bromides using (Ph_3_P)_3_CoCl.

Despite the evident versatility of Ni(CO)_4_ in synthesis, its toxicity has prompted the search for alternative methodologies. Perhaps the most useful reagent as a replacement is chlorotris(triphenylphosphine)cobalt(I), (Ph_3_P)_3_CoCl, readily preparable as an air-stable crystalline solid. Typically, its use in the coupling of allyl halides has been exemplified in a report by the Yamada group in Tokyo [21]. As illustrated in Scheme 14, the reaction of geranyl and farnesyl halides, **28** and **29**, respectively, and also of their geometrical isomers, **30** and **31**, with (Ph_3_P)_3_CoCl in benzene gave the corresponding geometrically pure coupling products **32**–**35**, under mild and non-basic conditions. This method provided a convenient route to squalenes.

**Scheme 15:**
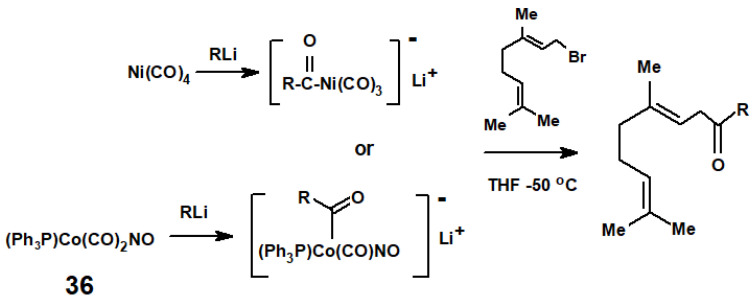
Use of (Ph_3_P)Co(CO)_2_NO as a replacement for Ni(CO)_4_.

In similar vein, nucleophilic attack by an alkyllithium on a carbonyl ligand of Ni(CO)_4_ leads to alkylnickel carbonylates that react with allyl halides, such as geranyl bromide, to yield ketones. However, with the aim of avoiding the use of such a toxic material as nickel tetracarbonyl, Hegedus and Perry sought to develop a safer and more convenient reagent that could bring about the same transformations. Isoelectronic cobalt nitrosyl tricarbonyl, Co(CO)_3_NO, is also a volatile, toxic liquid, but it reacts with triphenylphosphine to form (Ph_3_P)Co(CO)_2_NO, **36**, an orange, air-stable, non-volatile crystalline solid that is readily prepared and easily handled; gratifyingly, it also behaves as an efficient acylating agent (Scheme 15) [22].

## 4. Organoiron Complexes as Coupling Reagents

### 4.1. Oxaallyl-Fe(II) Intermediates

**Scheme 16:**
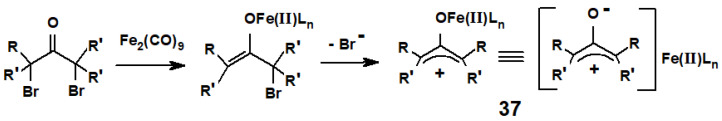
Formation of oxaallyl-Fe(II) intermediates from α,α′-dibromo ketones.

Ryōri Noyori (Nobel 2001) discovered that diiron enneacarbonyl, Fe_2_(CO)_9_—which slowly dissociates in solution to form Fe(CO)_5_ and the reactive coordinatively unsaturated moiety Fe(CO)_4_—reacts with α,α′-dibromo ketones to form oxaallyl-Fe(II) intermediates, **37**, that behave as allyl cations since the negative charge is masked by complexation with the Fe(II) ion (Scheme 16) [23].

**Scheme 17:**
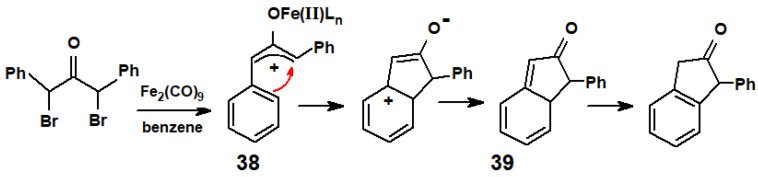
Electrocyclisation of the oxaallyl-Fe(II) species to form 1-phenyl-2-indanone.

These species undergo cycloadditions of the type “3 + 2 → 5”; typically, the reaction of Fe_2_(CO)_9_ with 1,3-dibromo-1,3-diphenylpropan-2-one yields 1-phenyl-2-indanone (70%). The proposed mechanism (Scheme 17) invokes nucleophilic attack on the allyl cation, **38**, by the adjacent aromatic ring to form **39**, that in turn undergoes aromatisation to yield the final product [24,25].

**Scheme 18:**
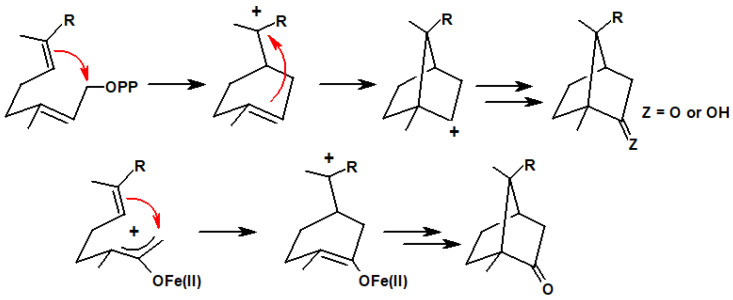
Proposed iron carbonyl-mediated route to camphor.

**Scheme 19:**
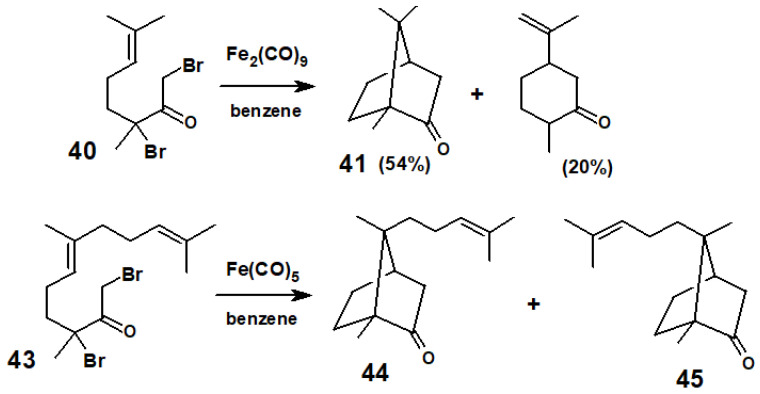
Noyori’s syntheses of (±)-camphor, **41**, and (±)-campherenones **44** and **45**.

Inspired by a biogenetic hypothesis for the formation of camphor, Noyori envisaged an iron carbonyl-mediated analogous reaction sequence (Scheme 18). Gratifyingly, when the dibromo ketone **40** (obtained from geraniol) and Fe_2_(CO)_9_ were heated in benzene in a pressure vessel, the major products were (±)-*camphor*, **41**, and (±)-*dihydrocarvone*, **42**, as shown in Scheme 19. Similarly, treatment of the dibromo ketone **43** (prepared from farnesol) and Fe(CO)_5_ delivered a 2:1 mixture of (±)-*campherenone*, **44**, and (±)-*epicampherenone*, **45**, in a 58% yield. Further examples of other natural product syntheses using this approach are given in a review by Noyori and Hayakawa [26].

### 4.2. Allyl-Iron(Dicarbonyl)(η^5^-Cyclopentadienyl) Complexes

**Scheme 20:**
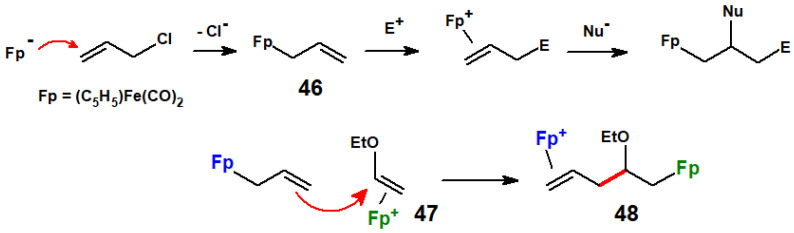
Interchange of σ- and π-bonded Fp environments.

A very different route to terpenoids, reported by Celebuski and Rosenblum [27], is based on the unique ability of (η^1^-allyl)Fp complexes, **46**, where Fp = (C_5_H_5_)Fe(CO)_2_, to flip easily and reversibly between monohapto (σ) and dihapto (π) bonding modes. Molecules of this type react with electrophiles such as the π-complexed ethyl vinyl ether, **47**, to yield the coupled product **48**; in this example, the σ-bonded and π-bonded Fp moieties have exchanged bonding modes (Scheme 20).

**Scheme 21:**
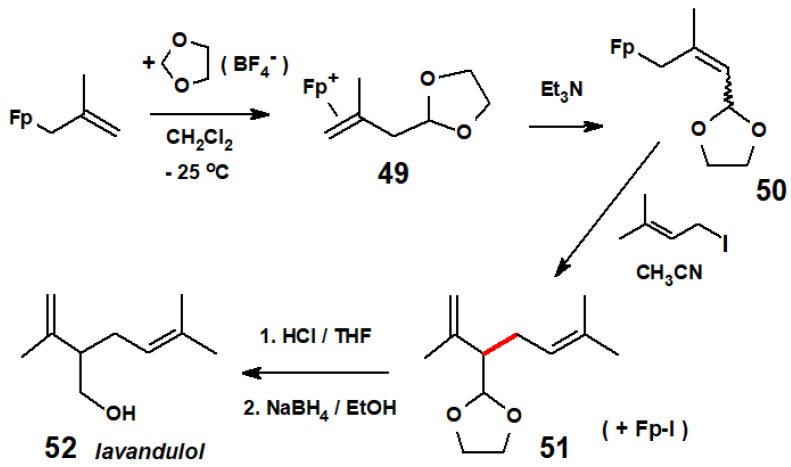
Synthesis of (±)-lavandulol mediated by the changeable hapticity of the (C_5_H_5_)Fe(CO)_2_, (Fp) group.

**Scheme 22:**
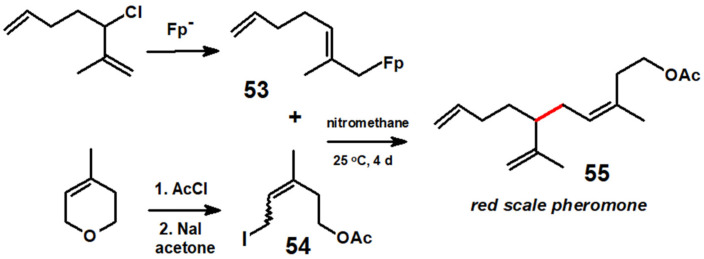
Synthesis of the (*R*,*S*)-California red scale pheromone, **55**.

This methodology has been exploited in the syntheses of terpenoids, as exemplified in Scheme 21 and Scheme 22. (Isobutenyl)Fp reacts with dioxolenium tetrafluoroborate to form **49** that, when deprotonated, yields the geometric isomers of **50** essentially quantitatively. Subsequent treatment with prenyl iodide delivers the ethylene acetal **51**; hydrolysis and reduction with sodium borohydride yields (±)-*lavandulol*, **52**, which is found in lavender oil, and whose *R*-enantiomer has a lemon-like or citrus fruit odour, and is used in some perfumes.

A somewhat more challenging target was a pheromone of the California red scale insect, a major pest of citrus fruit, that requires bringing together 1-Fp-2-methyl-2,6-heptadiene, **53**, and 1-iodo-3-methyl-5-acetoxy-2-pentene, **54**, preparable by the ring-opening of 5,6-dihydro4-methyl-2*H*-pyran, to form the desired product, **55**.

## 5. Palladium Complexes as Coupling Reagents

Palladium-mediated carbon–carbon coupling procedures are now a standard weapon in the synthetic chemist’s armoury. Many researchers made very significant contributions, but the Nobel regulations limit the number of chemistry laureates in a single year to three, and Richard Heck, Ei-ichi Negishi and Akira Suzuki all shared the award in 2010.

### 5.1. The Heck Reaction

**Scheme 23:**
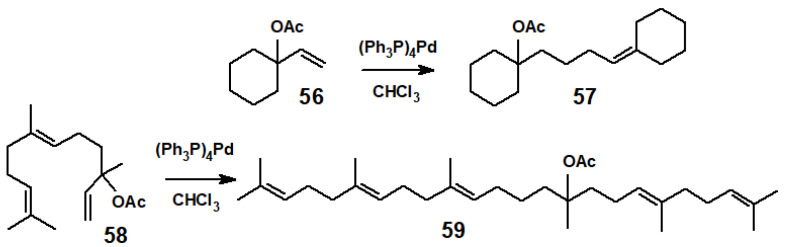
Dimerisation of 1-vinylcyclohexyl acetate and nerolidyl acetate by (Ph_3_P)_4_Pd.

Following a report by Heck in 1968 that palladium(II) salts induced coupling between allylic halides and arylmercurials [28], in 1970, Francis McQuillin at the University of Newcastle, U.K., found that simple monoterpenes underwent dimerisation when treated with tetrakis(triphenylphosphine)palladium(0) in chloroform, and extended this to its reaction with 1-vinylcyclohexyl acetate, **56**, to form 1-(4-cyclohexylidenebutyl)cyclohexyl acetate, **57** (Scheme 23). Moreover, the same reaction with nerolidyl acetate, **58**, also yielded a dimer, tentatively identified as **59**, the acetate derivative of squalene [29].

**Scheme 24:**
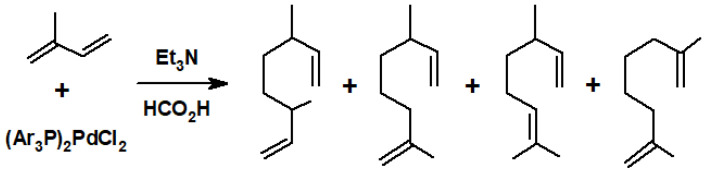
Palladium-catalysed dimerisation of isoprene.

**Scheme 25:**
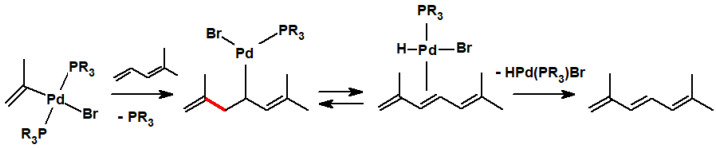
Vinylation of 4-methyl-1,3-pentadiene using Heck’s catalytic methodology.

Shortly thereafter, Heck reported that isoprene was dimerised when treated with complexes of the type (Ar_3_P)_2_PdCl_2_ in the presence of triethylamine and formic acid to yield a number of isomeric products (Scheme 24) in various ratios depending on the identity of the phosphine, the reaction time, and the solvent [30]. In addition, it was found that (Ar_3_P)_2_Pd(OAc)_2_ catalysed the vinylation of 4-methyl-1,3-pentadiene via the sequence depicted in Scheme 25 [31].

### 5.2. Prenylations and Geranylations Using π-Allyl-Palladium Complexes

A major breakthrough was the development of the Trost–Tsuji approach [32], whereby nucleophilic attack on a π-allyl-palladium complex brings about carbon–carbon coupling under stereochemical control [33,34]. Application to the synthesis of complex natural products has been comprehensively reviewed not only by Fairlamb and colleagues [35], but also by Barry Trost himself [36,37].

**Scheme 26:**
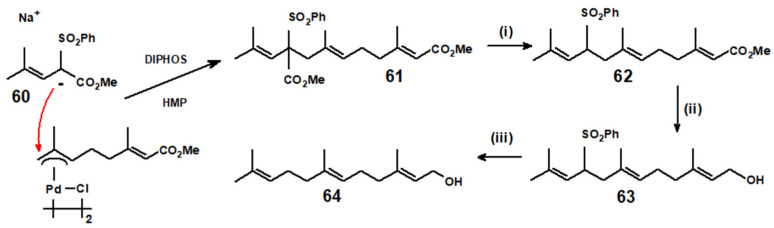
Trost’s prenylation route to (*E*,*E*)-farnesol: (i) LiI.3H_2_O, NaCN,DMF, 120 °C; (ii) DIBAL, toluene, −40 °C, then Na_2_SO_4_; (iii) Li, BrCH_2_CH_2_Br, −78 °C.

**Scheme 27:**
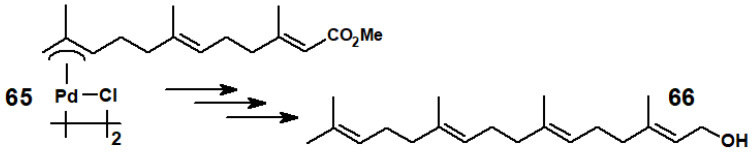
Trost’s prenylation route to (*E*,*E*,*E*)-geranylgeraniol.

The success of this approach is based on the preference for (η^3^-allyl)palladium complexes to form non-conjugated double bonds, and the ability of sulphur-stabilised carbanions to attack almost exclusively at the less-substituted allyl terminus. We focus here on prenylation, as exemplified in Scheme 26, in which the monoterpenoid methyl geraniate is converted into the sesquiterpenoid (*E*,*E*)-farnesol and then into the diterpenoid (*E*,*E*,*E*)-geranylgeraniol. Nucleophilic attack by the stabilised anion, **60**, leads to **61**, that is sequentially decarbomethoxylated, to form **62**, the terminal ester functionality reduced to the alcohol **63**, and finally desulphonylated to yield (*E*,*E*)-farnesol, **64**. As shown in Scheme 27, the analogous sequential procedure, but now starting from methyl farnesoate to form the allyl-palladium complex **65**, delivered (*E*,*E*,*E*)-geranylgeraniol, **66** [38].

**Scheme 28:**
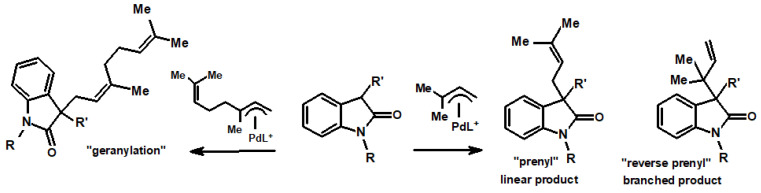
Geranylation, and linear-versus-branched prenylations.

We note, however, that later work by Trost revealed that “reverse prenylation” (Scheme 28) can also be achieved depending on the choice of Pd catalyst, and on the solvent, where toluene favoured prenylation; but in dichloromethane, the branched isomer was strongly preferred. They also reported examples of direct geranylation [39].

**Scheme 29:**
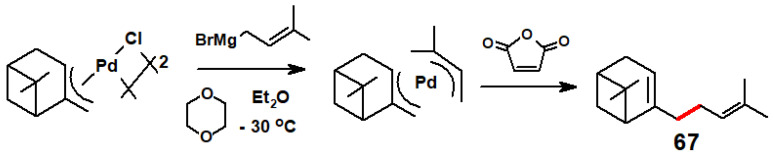
Head-to-head coupling induced by maleic anhydride.

In related work, Goliaszewski and Schwartz reported that allylic coupling arising from the reaction of bis(allylic)palladium species was facilitated by using π-acidic ligands, rather than phosphines [40]. Maleic anhydride was found to be effective in this regard, and the 1,5-dienic products formed were predominantly head-to-head adducts, as exemplified by the prenylation of pinene to form **67**, as shown in Scheme 29.

### 5.3. Palladium-Mediated Routes to Humulene, Grandisol, Vitamin A and Mokupalide

**Scheme 30:**
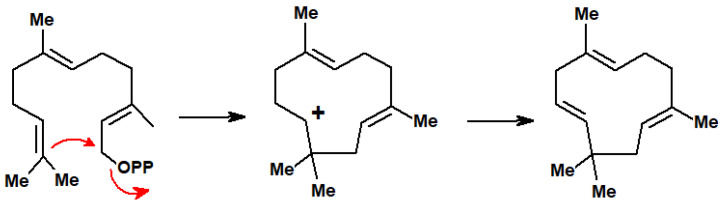
Biological route to humulene from farnesyl pyrophosphate.

**Scheme 31:**
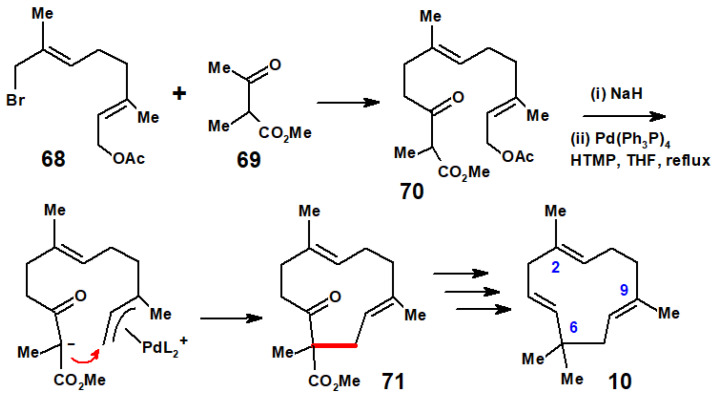
Principal steps in Nozaki’s route to humulene, **10**.

The first organometallic route to humulene, by Corey and Hamanaka, used Ni(CO)_4_ to couple two bromomethyl termini with the elimination of NiBr_2_ [12]. However, this same monocyclic sesquiterpene was also targeted by several groups using their newly developed palladium coupling techniques. Inspired by the biological formation of humulene via anti-Markovnikov cyclisation of farnesol pyrophosphate (Scheme 30), the Nozaki group at the University of Kyoto, Japan, designed a (π-allyl)palladium system poised to accept nucleophilic attack by a carbanion stabilised by a neighbouring ester functionality, as in Scheme 31. The *E*,*E*-bromide **68**, derived from geranyl acetate, reacted with the dianion of methyl α-methylacetoacetate, **69**, to form the keto ester **70** which, when deprotonated and slowly added to a solution containing (Ph_3_P)_4_Pd, led to the 11-membered ring system **71**; further manipulation furnished humulene [41].

**Scheme 32:**
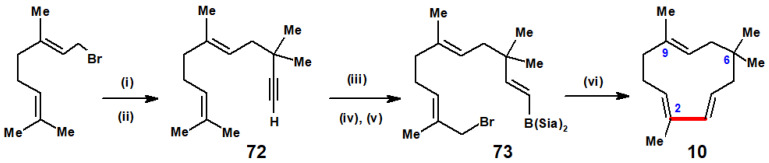
Suzuki’s route to humulene: (i) ClMg-CMe_2_-C≡C-SiMe_3_, (ii) KF, (iii) SeO_2_, EtOH, (iv) PBr_3_, Et_2_O, (v) HB(Sia)_2_, and (vi) Pd(PPh_3_)_4_, NaOH, benzene, reflux.

The approach to humulene taken by Akira Suzuki, not surprisingly, took advantage of his own methodology, whereby the terminal alkyne, **72**, was converted sequentially into the disiamylborane derivative **73** prior to palladium-mediated ring closure, as in Scheme 32 [42].

**Scheme 33:**
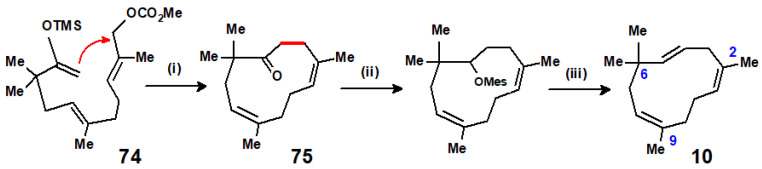
Synthesis of humulene by Hu and Corey: (i) Pd_2_(dba)_3_, dppf, THF 70 °C, 52% (ii) NaOH, i-PrOH, then MeSO_2_Cl, Et_3_N, 94%, and (iii) SiO_2_, CH_2_Cl_2_, 93%.

Yet, another short route to humulene was reported by Hu and Corey, whereby the allylic carbonate/silyl ether, **74**, underwent palladium coupling to form the cycloundecadienone **75**, as in Scheme 33. Subsequent mesylation and elimination delivered humulene in excellent yield [43].

**Scheme 34:**
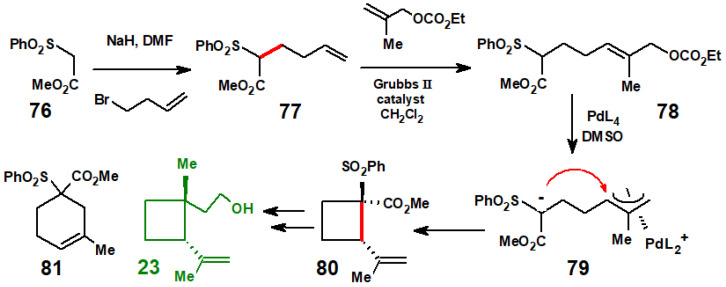
An allyl-palladium route to grandisol, **23**.

In 1973, the first organometallic route to grandisol, **23**, involved the nickel(0)-mediated coupling of isoprene prior to hydroboration [18]. Almost four decades later, the group led by Y-G. Suh at Seoul National University, Korea, reported a synthesis (Scheme 34) in which the anion of methyl benzenesulphonyl acetate, **76**, and 4-bromo-1-butene reacted to form **77**, that in turn underwent metathesis with ethyl (2-methylallyl) carbonate to yield **78**. Treatment with Pd(PPh_3_)_4_ in DMSO generated the allyl-palladium species, **79**, in which the sulphur-stabilised anion preferentially attacked intramolecularly in a 4-exo-trig fashion to form the desired cyclobutane ring, **80**. Further manipulation furnished grandisol [44]. The regioisomeric cyclohexene **81** was a minor side product.

**Scheme 35:**
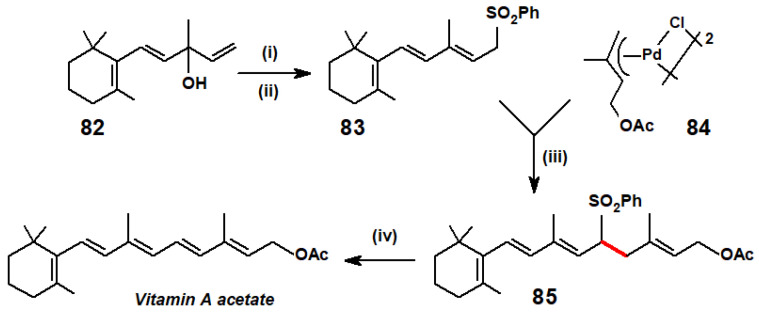
Allyl-palladium route to vitamin A acetate: (i) PBr_3_, (ii) PhSO_2_Na, (iii) NaH, PPh_3_, (iv) NaOEt, EtOH.

Manchand’s synthesis of vitamin A acetate in many ways resembles Trost’s procedure for the prenylation of methyl geraniate (Scheme 26). Starting from vinyl-β-ionol, **82**, bromination and introduction of the phenylsulphonyl group yielded **83**, the anion of which underwent nucleophilic attack on the allyl-palladium dimer, **84**, derived from prenyl acetate, to generate **85**. Desulphonylation completed the synthesis (Scheme 35) [45].

**Scheme 36:**
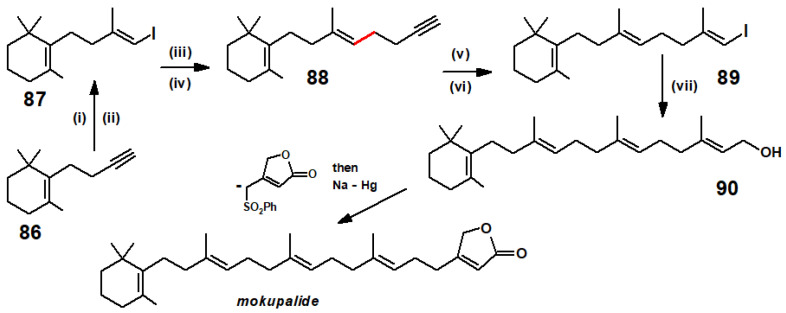
Negishi’s synthetic route to the tetraenol, **90**, and its subsequent conversion into mokupalide: (i) Me_3_Al—Cp_2_ZrCl_2_, CH_2_Cl_2_, (ii) I_2_, THF 0 °C, (iii) Me_3_SiC≡C-(CH_2_)_2_-ZnCl, Pd(PPh_3_)_4_, THF (iv) KF·2H_2_O, (v) Me_3_Al—Cp_2_ZrCl_2_, CH_2_Cl_2_, (vi) I_2_, THF 0 °C, (vii) n-BuLi, (CH_2_O)_n_.

An elegant example of a Pd coupling process facilitating the linking of multiple prenyl units was reported by Negishi [46], and is shown in Scheme 36. Starting from the terminal alkyne **86**, treatment with Me_3_Al—Cl_2_Zr(C_5_H_5_)_2_ and then with iodine generates the (*E*)-β-methyl-iodoalkene, **87**. Chain extension via palladium coupling with a homopropargylic zinc chloride to form **88**, followed by a second carbometallation and iodination, formed **89**. Finally, reaction with n-BuLi and paraformaldehyde led to the tetraenol **90**, which was subsequently converted by Sum and Weiler [47] into the C_30_ isoprenoid *mokupalide*, derived from a marine sponge.

## 6. Terpenoid Synthesis Using Titanium and Zirconium Reagents

### 6.1. Schwartz’s Hydrozirconation Reaction

In conjunction with allyl-palladium complexes, the cis addition of (C_5_H_5_)_2_Zr(H)Cl to terminal alkynes, thus generating vinylzirconium(IV) intermediates, reported by Schwartz [48], provides another convenient approach to carbon–carbon coupling. This concept has been exploited in the terpene field, notably in the elegant work of McMurry as exemplified in Section 6.2.

### 6.2. McMurry Dicarbonyl Coupling

**Scheme 37:**
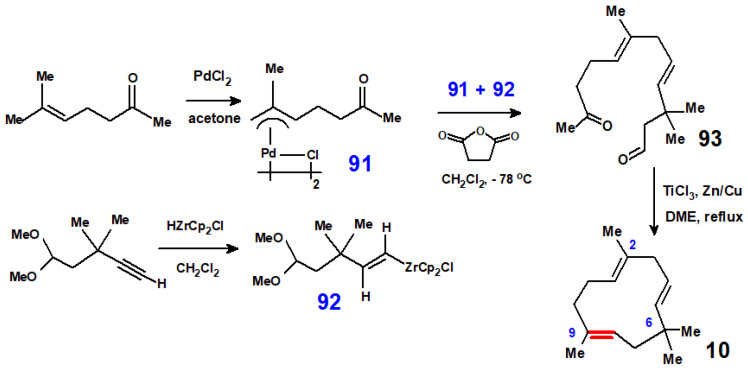
McMurry’s route to humulene.

The titanium-induced dicarbonyl coupling reaction devised by John McMurry at Cornell University, Ithaca, New York, brings about deoxygenative coupling and carbon–carbon double bond formation by reaction with TiCl_3_/Zn-Cu [49]. This has been brilliantly applied to terpenoid syntheses, and provides probably the most efficient organometallic route to humulene (Scheme 37). The required allyl-palladium precursor, **91**, was obtained by the reaction of commercially available 6-methyl-5-hepten-2-one with PdCl_2_ and was allowed to react with the vinyl zirconium complex, **92**, prepared by hydrozirconation of 5,5-dimethoxy-3,3-dimethyl-1-pentyne. The resulting ketoaldehyde, **93**, was subjected to McMurry coupling conditions to yield humulene in good yield in only four steps [50], an outstanding achievement!

**Scheme 38:**
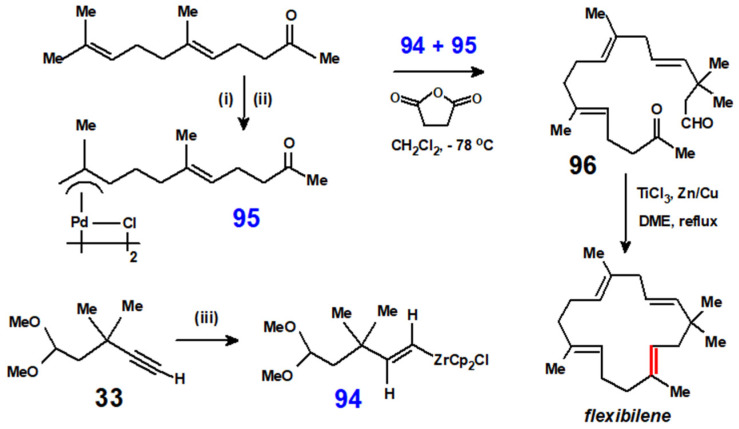
McMurry’s synthesis of flexibilene: (i) Pd(OCOCF_3_)_2_, acetone, (ii) Bu_4_NCl, (iii) HZrCp_2_Cl, CH_2_Cl_2_.

This same technique was utilised again to synthesise the first 15-membered ring diterpene to have been found in nature, *flexibilene*, 2,6,6,9,13-pentamethylcyclopentadeca-1,4,8,12-tetraene, that was originally isolated from the soft coral *Sinularia flexibilis*. Once again, hydrozirconation was used to obtain the required vinyl reagent, **94**, while its allyl-palladium counterpart, **95**, was prepared from geranyl acetone (Scheme 38). Under McMurry coupling conditions, the resulting keto-aldehyde, **96**, cyclised in a 78% yield [51], and the final product was characterised by comparison with an authentic sample.

**Scheme 39:**
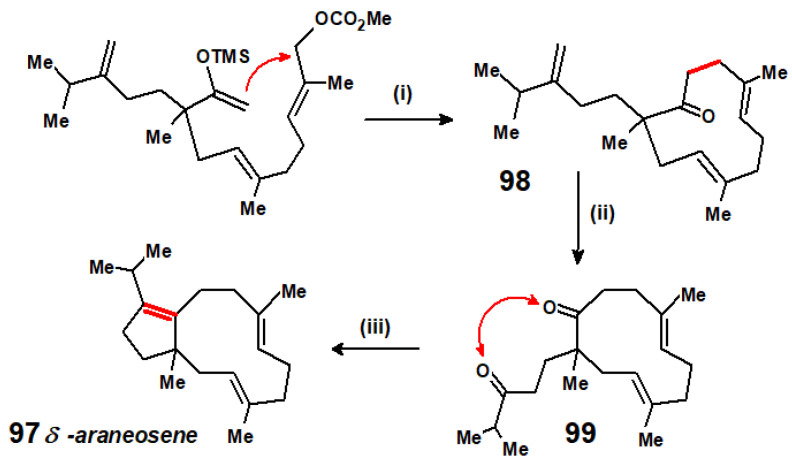
Final steps in the synthesis of (±)-δ-araneosene: (i) Pd_2_(dba)_3_, dppf, THF 70 °C, 48%; (ii) O_3_, CH_2_Cl_2_, Me_2_S, 54%; (iii) TiCl_3_, Zn-Cu, DME, 90%.

Another example of this efficient approach to ring-closing is given by the synthesis of (±)-δ-*araneosene*, **97**, by Hu and Corey that closely parallels their route to humulene, as shown previously (Scheme 33) [43]. The initial stages follow the palladium-mediated formation of the 11-membered ring with the difference that one of the methyl substituents at C(6) has been replaced by an extended chain bearing an isopropyl and a methylidene group, as in **98**. Ozonolysis of this latter functionality delivered the diketone **99** poised to undergo McMurry coupling and thereby yield the final product (Scheme 39).

**Scheme 40:**
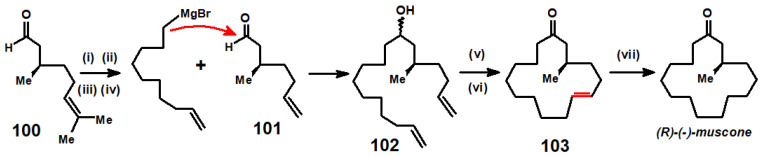
Ring-closing metathesis route to (*R*)-(-)-muscone, **26**, from (+)-citronellal: (i) NaBH_4_, MeOH, (ii) *t*-BuMe_2_SiCl, imidazole, DMF, (ii) OsO_4_, NaIO_4_, dioxane, (iii) Ph_3_PMeBr, *n*-BuLi, THF, −78 °C, (iv) SO_2_-pyridine, CH_2_Cl_2_, DMSO, Et_3_N, (v) Jones reagent, (vi) Grubbs II catalyst, (vii) H_2_, Pd-C.

An interesting parallel may be drawn between McMurry’s approach, whereby two oxygens are abstracted from adjacent carbonyl groups, thus leading to the formation of a carbon–carbon double bond and ring-closing metathesis (RCM), in which a pair of terminal alkenes can exchange partners and eliminate ethylene. In the terpene field, this latter methodology was used to advantage in an ingenious route to (*R*)-(-)-muscone [52]. Commercially available (+)-citronellal, **100**, was reduced to citronellol, protected as an ether with *t*-BuMe_2_SiCl, and then treated with OsO_4_/NaIO_4_ to cleave the isopropenyl linkage, followed by Wittig olefination to give **101**, which was allowed to react with the Grignard derived from 10-bromodec-1-ene to form alcohol **102**. After oxidation to ketone, RCM using the Grubbs II catalyst produced the 15-membered cycloalkene, **103**, that yielded (*R*)-(-)-muscone upon hydrogenation (Scheme 40).

### 6.3. Ti(III) and Zr(III) as Dimerisation Catalysts

**Scheme 41:**
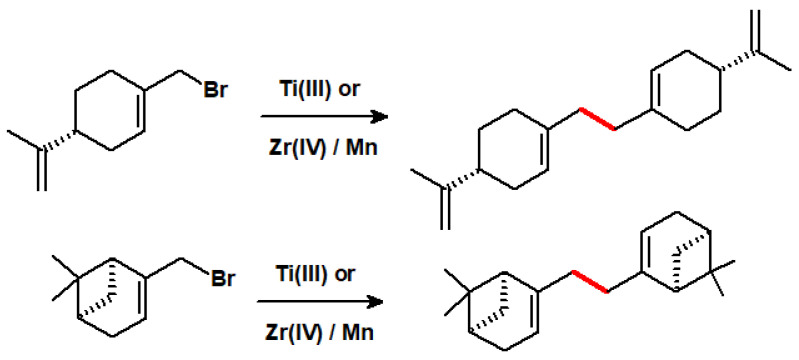
Terpene dimerisations induced by Ti(III) or Zr(III) catalysts.

Group 4′s metal-mediated coupling of allylic halides has been described by Barrero who found that a Ti(III) catalyst, prepared from titanocene dichloride and powdered manganese, efficiently brings about a number of dimerisation processes. The analogous Zr(III) system, made from Cp_2_ZrCl_2_ and Mn, behaves similarly and, interestingly, the addition of Lewis acids, such as collidine hydrochloride, allows catalyst loading to be as low as 0.05 equivalents [53]. Typical examples are shown in Scheme 41.

## 7. The Golden Route to Carenes

**Scheme 42:**
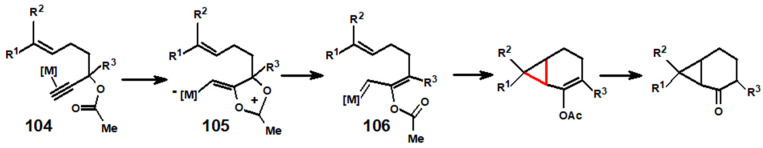
Proposed route to carenes via gold(III)-catalysed rearrangement of propargyl acetates.

While carene and its homologues are conventionally prepared via cyclopropanation of unsaturated ketones, in an elegant new approach, Fürstner and Hannen hypothesised that metal-catalysed rearrangement of a propargyl acetate to a metal carbene could provide a more concise synthetic protocol. As indicated in Scheme 42, starting from the alkyne-metal π-complex, **104**, migration of the acetate via a cyclic intermediate, **105**, to form the metal carbene complex **106** could then bring about cyclopropane formation [54]. The completion of the route to the carene skeleton would merely require a conversion of the acetate to ketone and an introduction of a double bond.

**Scheme 43:**
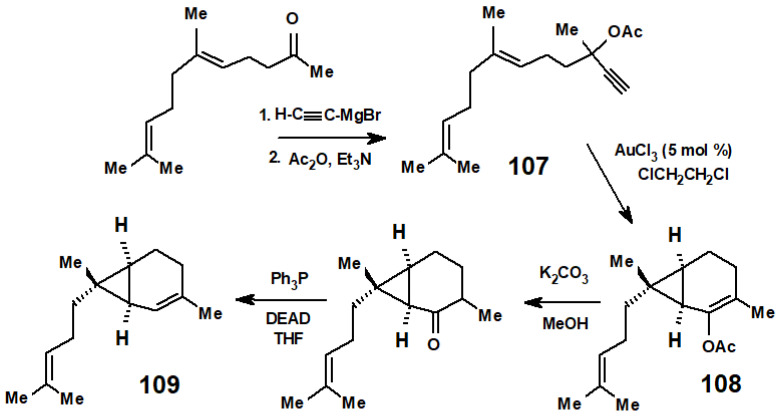
Gold-catalysed synthesis of sesquicarene, **109**.

Gratifyingly, when the propargyl acetate **107**, derived from geranylacetone, was treated with AuCl_3_ (Scheme 43), ring closure to **108** was successfully achieved in excellent yield and purity (95%). Further manipulation delivered (-)-*sesquicarene*, **109**.

## 8. Reactions of Terpene—Palladium(II) Complexes with Nucleophiles

**Scheme 44:**
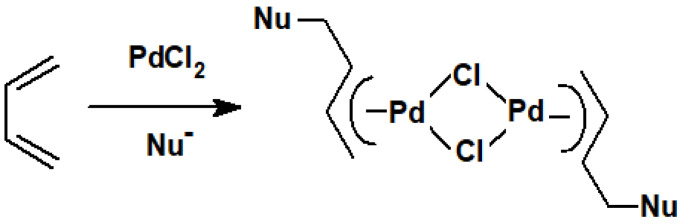
Addition of a nucleophile to a diene to form an allyl-palladium chloride dimer.

The preponderance of alkene double bonds in terpenoids inevitably suggested that treatment with complexes of palladium(II), iron(0), or nickel(0) would be the most productive areas to be investigated initially. The general reaction with a source of PdCl_2_ is to form a π-allyl-palladium chloride dimer in which the nucleophile has added to a terminal carbon of the original diene (Scheme 44).

In a series of publications in the 1970s, Francis McQuillin (in Newcastle, UK) and Hiroharu Suzuki (in Tokyo) studied the reactions of palladium chloride with the isomeric monoterpenes *ocimene* and *myrcene* that are found in numerous natural sources, such as bay leaves, hops or wild thyme, and are widely used in the perfume industry.

**Scheme 45:**
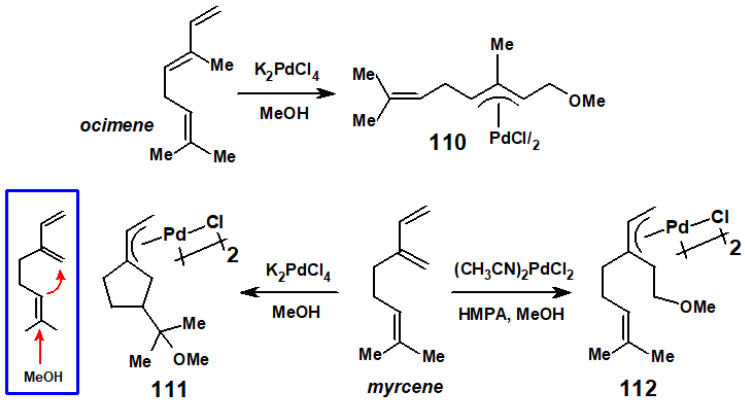
Reactions of ocimene and myrcene with methanol, induced by L_2_PdCl_2_.

Both *cis* and *trans* ocimene react with K_2_PdCl_4_ in methanol, bringing about a colour change from red-brown to yellow, to form the η^3^-palladium complex **110**, that was characterised principally by NMR spectroscopy and mass spectrometry. In contrast, the analogous reaction of myrcene in methanol yielded a product, **111**, comprising an exo-cyclic allyl-palladium fragment and a cyclopentane ring bearing a CMe_2_OMe substituent. The overall effect was to bring about palladium-promoted nucleophilic attack on the terminal carbon of the unconjugated double bond with concomitant cyclisation to form the five-membered ring, as depicted in Scheme 45 [55]. Changing the palladium precursor and the solvent to hexamethylphosphoramide led instead to **112**, as the primary product [56,57].

**Scheme 46:**
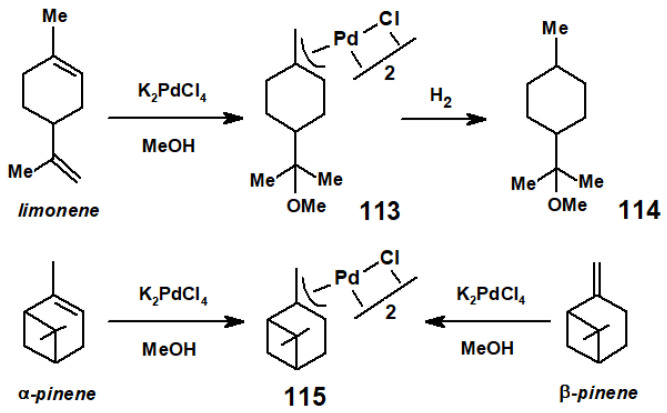
Reactions of limonene or pinene with potassium tetrachloropalladate(II).

The corresponding reaction of *limonene* in methanol solution proceeded via the exocyclic η^3^-palladium complex **113** that yielded dihydro-α-dihydroterpinyl methyl ether, **114**, upon hydrogenation, while palladation of either α- and β-*pinene* yielded the same π-allyl complex, **115** (Scheme 46) [58].

**Scheme 47:**
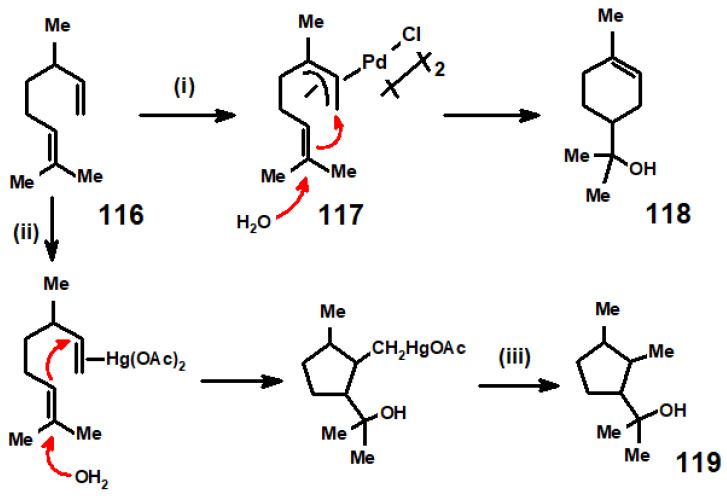
Reactions of 3,7-dimethylocta-1,6-diene, **116**, with palladium or mercury salts: (i) PdCl_2_-aqueous acetone, (ii) Hg(OAc)_2_, (iii) H_2_O-THF-NaBH_4_.

McQuillin also probed the products formed when (+)-3,7-dimethylocta-1,6-diene, **116**, was treated either with palladium dichloride or mercuric acetate in different solvents (Scheme 47) [59]. This isoprenoid diene is readily available from pyrolysis of (+)-pinane [2,60] and, when the reaction was carried out in acetone at 70 °C, cyclisation in the allyl-palladium complex **117** proceeded in Markovnikov fashion to yield the six-membered ring in α-*terpineol*, **118**.

By way of contrast, when 3,7-dimethylocta-1,6-diene was stirred with mercuric acetate in aqueous THF and then reduced with sodium borohydride, the products were reported to be stereoisomers of (2,3-dimethylcyclopentyl)propan-2-ol, **119** [59]. The proposed Hg(II)-promoted mechanism invokes anti-Markovikov addition to the coordinated double bond. As noted below, such molecules had been described previously as products of the thermal cyclisation of linalool, and named as iridanols [61].

**Scheme 48:**
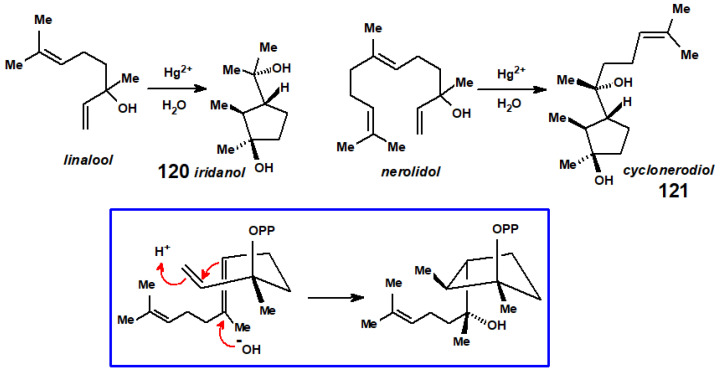
Cyclisation of linalool and of nerolidol mediated by a mercury(II) salt.

In very closely related work by the Itô group in Japan [62], also using a mercury(II) salt, the cyclisation of the monoterpene linalool to iridanol, **120**, or of the sesquiterpene (C_15_) system nerolidol to cyclonerodiol, **121**, proceeds with almost exclusive stereospecificity. In many ways, this mimics the biosynthetic pathway elucidated by Cane [63], as shown in the box in Scheme 48.

## 9. Reactions of Terpene—Iron Carbonyl Complexes with Electrophiles

**Scheme 49:**
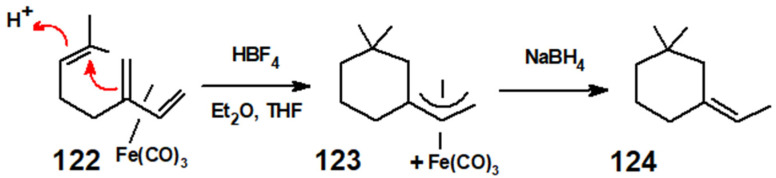
Protonation of (myrcene)Fe(CO)_3_ followed by reduction with NaBH_4_.

Myrcene reacts with Fe_2_(CO)_9_, in benzene, or better with Fe_3_(CO)_12_ in dibutyl ether, to form **122,** in which the tricarbonyliron fragment is η^4^-bonded to the conjugated diene unit. When protonated with HBF_4_ in ether, it brings about cyclisation and the formation of the allyl cation complex, **123**; as shown in Scheme 49, subsequent treatment with sodium borohydride yields 1-ethylidene-3,3-dimethylcyclohexane, **124** [64].

**Scheme 50:**
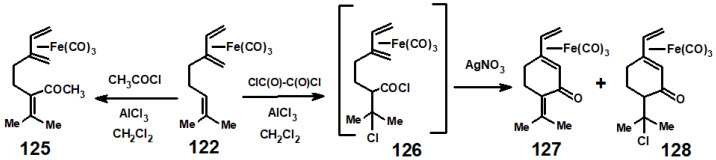
Carbonylative annulation of (myrcene)Fe(CO)_3_.

Treatment of **122** with acetyl chloride under Friedel–Crafts conditions brings about the introduction of an acetyl group on the isolated double bond, as in **125**. More interestingly, the use of oxalyl chloride led to the acyl chloride, **126**, which reacted with AgNO_3_ to yield the two cyclic ketone complexes, **127** and **128**, via carbonylative annulation (Scheme 50) [65].

**Scheme 51:**
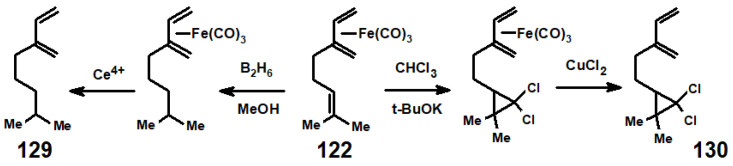
Reduction in the isopropylidene unit or addition of dichlorocarbene to (myrcene)Fe(CO)_3_.

The π-bonded Fe(CO)_3_ in **122** can also act as a protecting group whereby a reaction that would normally occur on the diene instead proceeds at the unconjugated alkene terminus. Typically, treatment with diborane reduces the isopropylidene unit, and decomplexation with ceric ammonium nitrate yields the dihydro monoterpene, **129** [66]. Likewise, addition of dichlorocarbene, generated from chloroform and potassium tert-butoxide, forms the dichloro-dimethylcyclopropane **130** after decomplexation (Scheme 51) [67].

**Scheme 52:**
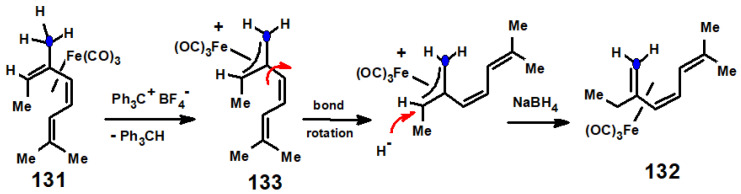
Proposed rearrangement mechanism of (ocimene)Fe(CO)_3_.

The reaction of (ocimene)Fe(CO)_3_, **131**, with trityl fluoroborate, followed by reduction with sodium borohydride, brings about a rearrangement such that the transfer of a hydrogen from the methyl group onto its ethylidene neighbour led to an isomer, **132**, bearing a methylene and an ethyl substituent. One can envisage the formation of the allyl cation, **133**, and then the bond rotation, thus offering the Fe(CO)_3_ unit a potential cisoid diene fragment available for complexation after migration as well as the addition of hydride to complete the formation of the new ethyl substituent. In support of this scenario, the original methyl group was doubly labelled with tritium and carbon-14, and the final positions of these labels were elucidated (Scheme 52) [66].

## 10. Metal-Promoted Terpene Isomerisations

### 10.1. π-Allyl Metal Hydrides

**Scheme 53:**
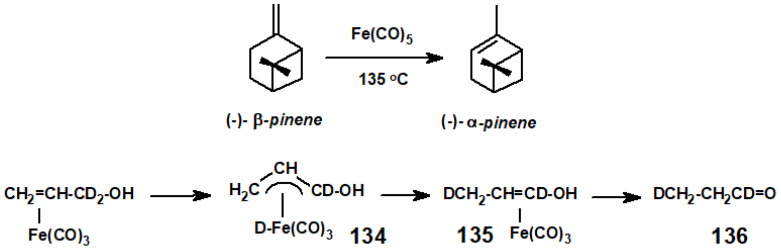
Evidence for a (π-allyl)tricarbonyliron hydride in an isomerisation process.

The observation of an increasing number of hydrogen rearrangement processes in alkenes prompted suggestions of the involvement of π-allyl metal hydrides [68]. Typically, (-)-β-pinene is converted into high optical purity (-)-α-pinene when heated with iron pentacarbonyl at 135 °C [69]. In support of this hypothesis, it was found that when the isotopically labelled allyl alcohol CH_2_=CH-CD_2_-OH was treated with Fe(CO)_5_, the NMR spectrum of the resulting propionaldehyde, CH_2_D-CH_2_-CD=O, revealed the presence of deuterium in the methyl but *not* in the methylene group. Scheme 53 illustrates the mechanism by which the intermediate **134** allows the transfer of deuterium, leading to the vinyl alcohol–iron carbonyl, **135**, that tautomerizes upon decomplexation to yield the labelled propionaldehyde, **136** [70].

**Scheme 54:**
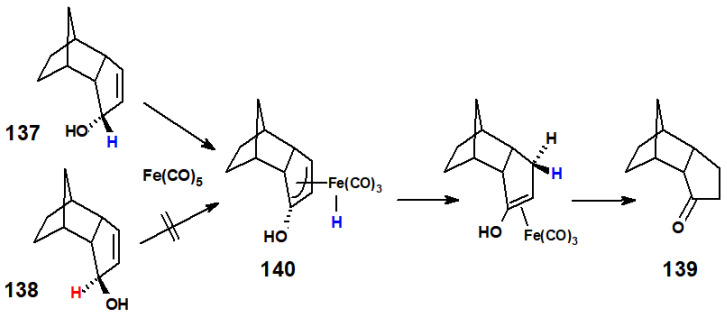
Reactions of *exo* and *endo* 1-hydroxydicyclopentadiene with Fe(CO)_5_.

This work was later extended, whereby *endo* and *exo* 1-hydroxy-5,6-dihydrodicyclopentadiene, **137** and **138**, respectively, were each heated with Fe(CO)_5_ in attempts to bring about isomerisation to the ketone, **139**. In the event, only the *endo*-hydroxy epimer, **137**, underwent exchange, indicating that hydrogen migration via the *exo* (π-allyl)tricarbonyliron hydride, **140**, was only viable when the iron was complexed to the same face as the migrating hydrogen (Scheme 54) [71]. Examination of models clearly shows that *endo* coordination of the bulky tricarbonyliron fragment would encounter serious steric problems.

**Figure 3:**
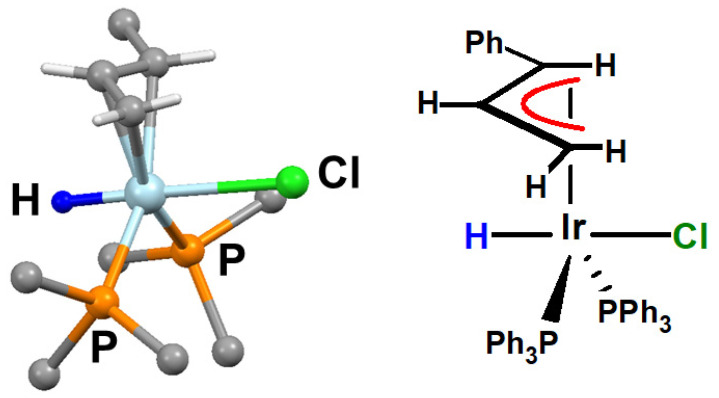
Molecular structure of the (π-allyl)iridium hydride, **141**; for clarity, only the *ipso* carbons of the phenyl rings are shown.

Analogous rearrangements involving other π-allyl-metal hydride intermediates are also known and, gratifyingly, the structure of the allyl iridium hydride complex IrClH(η^3^-C_3_H_4_Ph)(PPh_3_)_2_, **141**, has been unambiguously determined by X-ray crystallography (Figure 3) [72].

### 10.2. [3,3]-Sigmatropic Shifts in Terpenoids

**Scheme 55:**
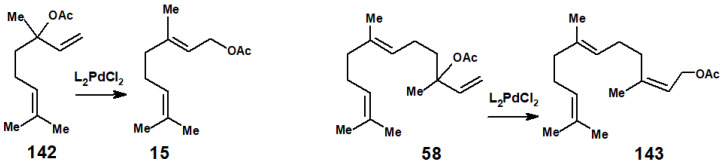
1,3-Migrations in linalyl and nerolidyl acetates.

**Scheme 56:**
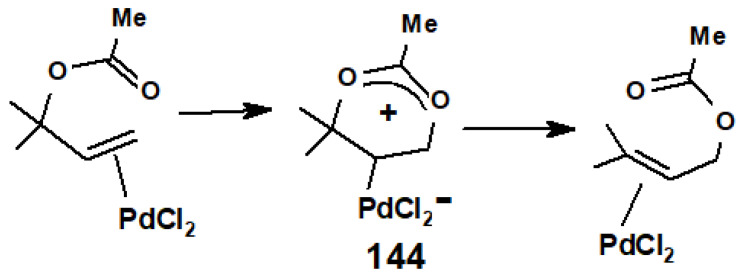
Proposed mechanism of a PdCl_2_-initiated 1,3-acetyl rearrangement.

As part of his early pioneering work, McQuillin studied the rearrangement behaviour of *linalyl* and *neronidyl* acetates, **142** and **58**, respectively, when allowed to react with K_2_PdCl_4_ in chloroform in the presence of calcium carbonate (acting as a buffer to prevent acid-induced reactions). It was found that in both cases, the acetate underwent a 1,3-migration to form their *geranyl* and *farnesyl* isomers, **15** and **143** (Scheme 55), and a mechanistic proposal invoked the formation of a cyclic palladium-linked bridging acetate intermediate, **144** (Scheme 56) [58].

**Scheme 57:**
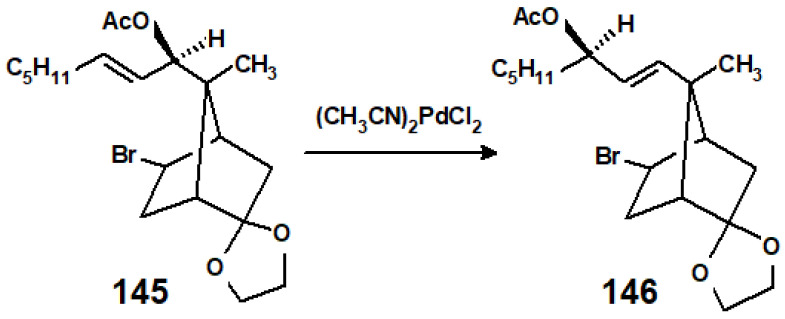
A 1,3-acetate migration with complete transfer of chirality.

A particularly fine example, also in the terpenoid field, was reported, whereby the 1,3-acetate shift in the prostaglandin precursor **145** proceeded to yield **146** in a suprafacial fashion with a complete transfer of chirality (Scheme 57) [73].

**Scheme 58:**
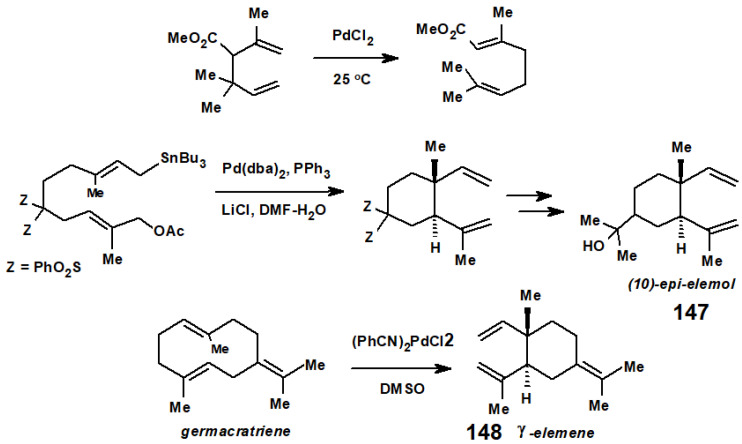
Examples of Cope rearrangements in terpenoids.

These can now be classified as palladium-initiated [3,3]-sigmatropic shifts, and selected examples of Cope rearrangements in terpenoids [74,75,76,77], including synthetic routes to (10)-*epi-elemol*, **147** [78], and γ-elemene, **148** [79], are shown in Scheme 58. Many such metal-promoted rearrangements proceed under very mild conditions, presumably via the coupling of π-allyl intermediates, whereas their non-complexed counterparts frequently require prolonged heating.

### 10.3. Interactions of Ring-Strained Terpenes with Organometallic Reagents

**Scheme 59:**
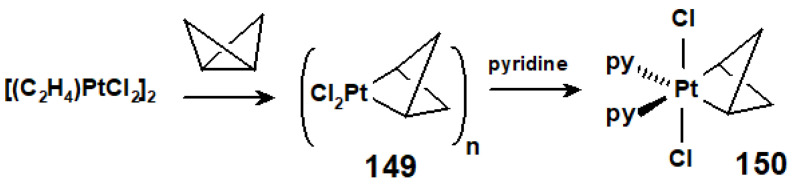
Ring-opening of bicyclobutane by a Pt(II) complex.

The tendency of many organometallic species to bring about ring-opening in strained systems is well established. Typically, the reaction of bicyclobutane with a platinum(II) complex possessing a readily displaceable ethylene ligand results in the formation of a polymeric intermediate, **149**, that reacts with pyridine to yield **150** (Scheme 59) [80].

**Scheme 60:**
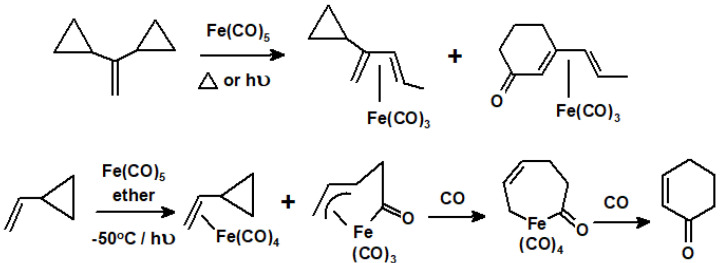
Ring-opening of cyclopropyl substituents by reaction with Fe(CO)_5_.

Similarly, when vinylcyclopropanes are thermolyzed or photolyzed with Fe(CO)_5_, they suffer ring-opening to yield diene-Fe(CO)_3_ complexes, and can also lead to carbonyl insertion products, as exemplified in Scheme 60 [81,82].

**Scheme 61:**
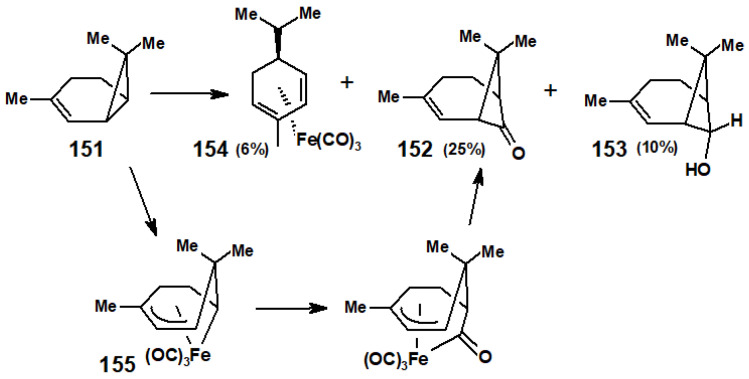
Ring-opening of the cyclopropyl moiety in (+)-2-carene by Fe(CO)_5_.

Such behaviour is also found in the terpene field, whereby thermolysis of (+)-2-carene, **151**, and Fe(CO)_5_ yields (-)-(1*S*)-3,8,8-trimethylbicyclo[4.1.1]oct-3-ene-7-one, **152**, and the corresponding alcohol, **153**, along with (α-phellandrene)Fe(CO)_3_, **154** [83]. The proposed mechanism (Scheme 61) invokes cleavage of the bond common to the 3-membered and 6-membered rings with the formation of the allyl-iron intermediate, **155**, the migratory insertion of a carbonyl, and finally, the reductive elimination to form the ketone **152**. The alcohol **153** is thought to arise from reduction of **152** by H_2_Fe(CO)_4_.

**Scheme 62:**
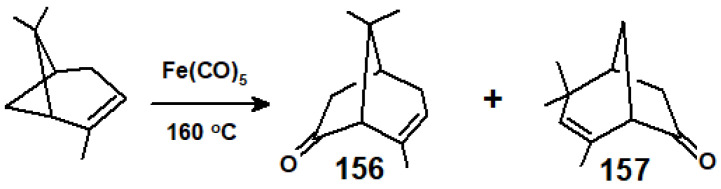
Reaction of pinene with Fe(CO)_5_ to bring about carbonyl insertion.

In earlier work on the somewhat less-strained (+)-(α)-pinene system, prolonged thermolysis with Fe(CO)_5_ in a pressure vessel at 160 °C led to two stereospecific ring expansion products, **156** and **157**, in which the bicyclo[3.2.1] molecular frameworks were enantiomeric, but with differently positioned methyl substituents (Scheme 62) [84].

**Scheme 63:**
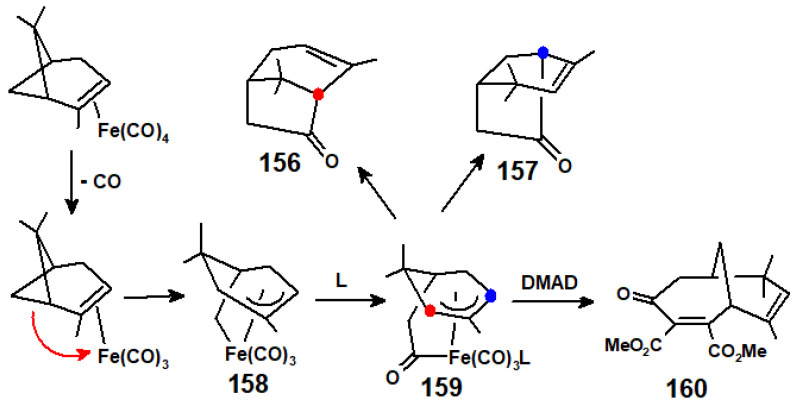
Proposed mechanism of ring expansion of pinene induced by Fe(CO)_3_.

The proposed mechanism involved the cleavage of the cyclobutane ring to form an intermediate, **158**, possessing an Fe(CO)_3_ fragment bonded in both in a π-allyl- and a σ-alkyl fashion. Subsequent carbonyl insertion furnished **159** that was exquisitely poised for reductive elimination to occur with concomitant C-C bond formation at either end of the allyl unit, leading to **156** and **157** (Scheme 63). Moreover, further reaction of the enantiomer of **159**, derived from (-)-(α)-pinene, with dimethyl acetylenedicarboxylate (DMAD) brings about an insertion of the alkyne to deliver the bicyclo[4.3.1]octadienone, **160** [85].

**Figure 4:**
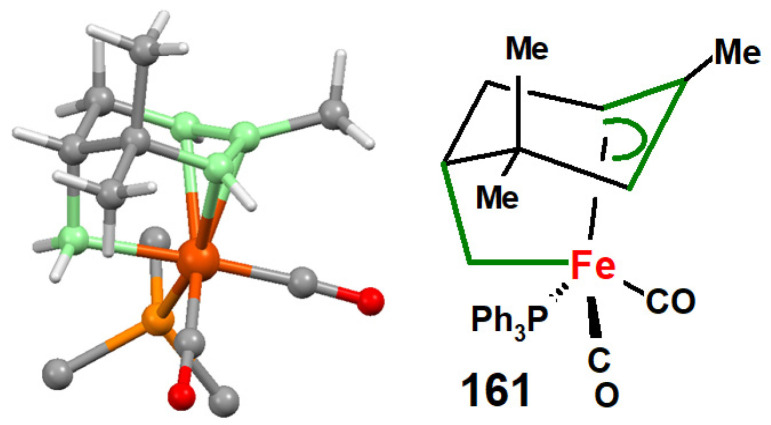
Molecular structure of **161,** showing the π-allyl/σ-alkyl bonding to iron; for clarity, only the ipso carbons of the Ph_3_P ligand are shown.

Impressively, in 1975, the authors characterised their products and suggested a reaction mechanism solely on the basis of their NMR data (at 60 MHz) and mass spectra [85]. Gratifyingly, their proposals were fully vindicated almost three decades later by the isolation and X-ray crystallographic structural determination of the π-allyl/σ-alkyl intermediate, **161** (Figure 4) [86].

### 10.4. Terpene Rearrangements Induced by Pt(II) or Rh(I) Salts

**Scheme 64:**
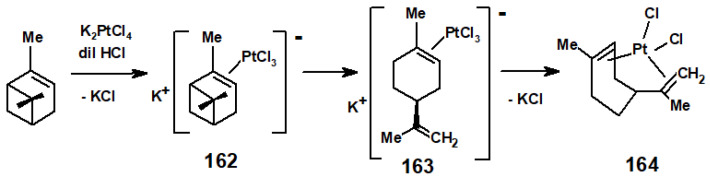
Ring-opening of pinene by K_2_PtCl_4_.

Pinene is also ring-opened when left in solution at room temperature with K_2_PtCl_4_ in dilute HCl for an extended period [87]. The product isolated and characterised by X-ray crystallography was found to be dichloro[η^4^-p-mentha-1,8(9)-diene]platinum(II) in which the metal is coordinated not only to the original double bond in pinene, but also to the isopropenyl group formed upon ring cleavage. The mechanism envisaged (Scheme 64) requires initial coordination to pinene, as in **162**, ring-opening to form **163**, and loss of KCl to yield the observed product **164**.

**Scheme 65:**
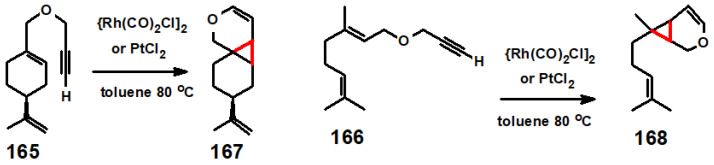
Cyclopropane formation induced by Rh(I) or Pt(II) salts.

**Scheme 66:**
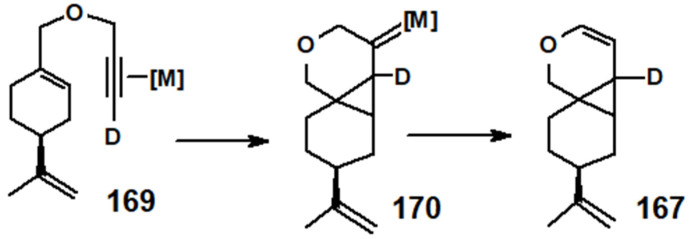
Proposed mechanism for cyclopropane formation via a metal carbene intermediate.

The introduction of a terminal alkyne substituent, thus forming a terpenoid enyne, led to unexpected cyclopropane formation when treated with a Rh(I) or Pt(II) salt. The enynes **165** and **166**, derived from perillyl alcohol and geraniol, respectively, reacted to form the cyclopropyl systems **167** and **168** shown in Scheme 65. The favoured mechanism invokes complexation to the alkyne, as in **169**, a rearrangement to a metal carbene complex, **170**, with subsequent addition to the neighbouring double bond to form the cyclopropane, and is supported by isotopic labelling, as shown in Scheme 66 [88]. In many ways, this parallels the approach towards cyclopropane formation taken by Fürstner in his gold-carbene route to carenes (Section 7).

## 11. Regiospecific Epoxidation of Terpenes

**Scheme 67:**
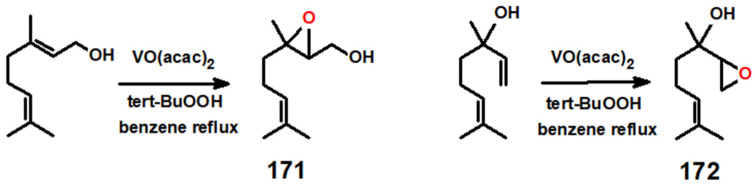
Catalytic selective epoxidation of (left) geraniol and (right) linalool.

Epoxidation is frequently accomplished by the oxidisation of an alkene linkage by a peroxy acid. However, when the target molecule has multiple sites of unsaturation, stereo- and regio-selectivity need to be controlled. This was first achieved in the terpene field by K.B. Sharpless (Nobel 2001, 2022), whereby geraniol and linalool were selectively converted into the previously unknown epoxides, **171** and **172**, respectively, using vanadyl acetylacetonate as the catalyst in conjunction with tert-butyl hydroperoxide (Scheme 67) [89]. In contrast, reaction with meta-chloro-perbenzoic acid is markedly less selective. The Sharpless approach has since been very widely adopted, and it is interesting to note that almost half a century later, it was reported that, when this catalyst was grafted onto silica nanoparticles, 2,3-epoxygeraniol was obtained with 100% conversion and 99% selectivity [90].

**Scheme 68:**
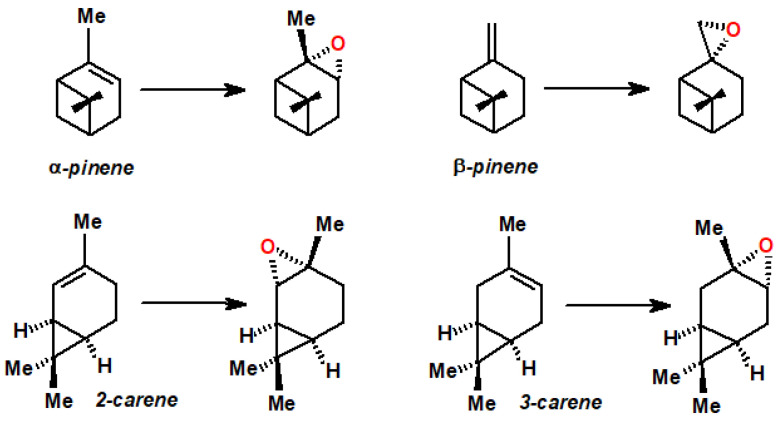
Catalytic selective epoxidation of pinenes and carenes.

In another early example, the combination of *tert*-amyl hydroperoxide and either molybdenum hexacarbonyl or molybdenum pentachloride brought about specific and almost quantitative conversion of α- and β-pinenes or 2- or 3-carenes into their corresponding epoxides (Scheme 68) [91].

**Scheme 69:**
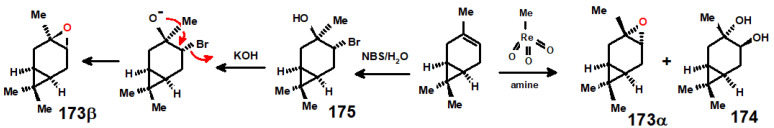
Stereospecific epoxidation of (+)-3-carene with methyltrioxorhenium(VII).

In closely related later work [92], the reaction of (+)-3-carene with the high oxidation state complex methyltrioxorhenium(VII), in the presence of a Lewis base, led to stereospecific, almost quantitative, formation of α-3,4-epoxycarene, **173α**, along with the diol **174**. Interestingly, isomeric β-3,4-epoxycarene, **173β**, was preparable by the treatment of (+)-3-carene with N-bromosuccinimide and water to deliver the bromohydrin, **175**, with subsequent epoxide formation, resulting from base-promoted elimination of HBr (Scheme 69).

**Scheme 70:**
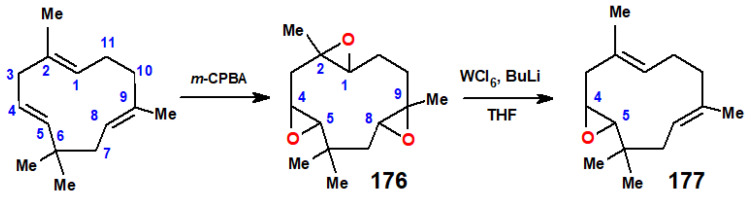
Synthetic route to humulene-3,4-epoxide, **177**.

**Figure 5:**
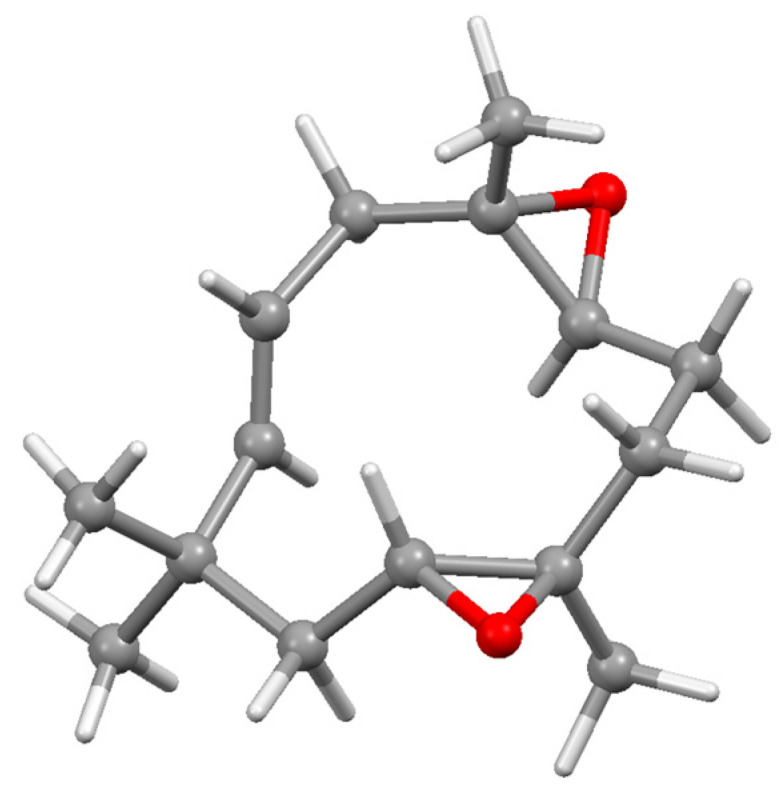
Molecular structure of humulene-(1,2)(8,9)-di-epoxide.

Epoxidation of humulene posed a particular problem when there was a need to functionalise only at C(3)–C(4), the least reactive double bond, because epoxidation occurs preferentially at C(1)–C(2), the most strained alkene linkage, and then at C(8)–C(9), as shown in the X-ray crystal structure of the di-epoxide (Figure 5) [93]. This difficulty was finally overcome [94] by first generating the tri-epoxide, **176**, by continued treatment with *m*-chloroperbenzoic acid, and then removing the oxygens at C(1)–C(2) and C(8)–C(9) by reaction with WCl_6_ and *n*-BuLi, a deoxygenation reaction previously discovered by Sharpless [95], to form **177** (Scheme 70).

Attempts to attach organometallic fragments to humulene were generally unsuccessful, although there is a report of the formation of the π complex [(humulene)Fe(CO)_2_(C_5_H_5_)]^+^ [BF_4_]^-^; however, the combination of low yield and thermal instability precluded its unambiguous identification by NMR spectroscopy [96].

## 12. Organometallic Derivatives of Guaiazulene

**Scheme 71:**
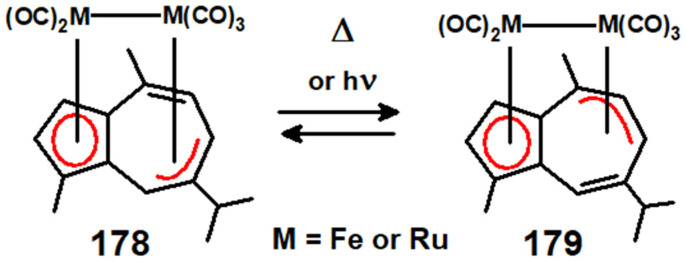
Migration of one component of a bimetallic fragment across the guaiazulene framework.

Guaiazulene (1,4-dimethyl-7-isopropylazulene) is a dark blue bicyclic sesquiterpene possessing five- and seven-membered rings, found as a constituent in oil of guaiac and in chamomile oil, and is widely used in skin care products. The synthesis and structure of the many metal complexes of azulene itself has been comprehensively reviewed [97]. Early work by Cotton established that guaiazulene forms bimetallic complexes in which the linked metals bind in a pentahapto fashion to the 5-membered ring and in a trihapto mode to the 7-membered ring (Scheme 71) [98]. Unlike azulene with its *C*_2v_ symmetry, the pattern of substituents in guaiazulene breaks the mirror orthogonal to the molecular plane, thus reducing its symmetry to *C*_S_, a single mirror plane. Now, complexation of one metal (or more) to one face of the molecule lowers the symmetry to *C*_1_ and renders the system chiral. Moreover, these molecules can adopt two different trihapto coordination sites, as in **178** and **179**, which have been synthesised, separated chromatographically and then fully characterised by X-ray crystallography. Variable-temperature NMR data reveal that these coordination isomers interconvert when heated. Later studies established that this interconversion can also be induced photochemically, and the barrier to haptotropic migration was found to be 117 ± 8 kJ mol^−1^ [99]; in the ruthenium analogues, the barriers were found to be markedly higher [100].

**Figure 6:**
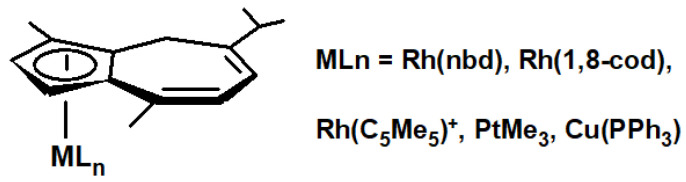
Selected organometallic derivatives of the dihydroguaiazulenide anion.

In an excellent very recent publication, it was reported that hydrolithiation of guaiazulene yields the isomerically pure dihydroguaiazulenide anion, a stable and storable cyclopentadienide-type ligand ideally suited for reaction with a wide range of organometallic precursors [101]. A number of half-sandwich complexes shown in Figure 6, in particular the trimethylplatinum complex, exhibit excellent catalytic activity in alkene hydrosilylations.

**Figure 7:**
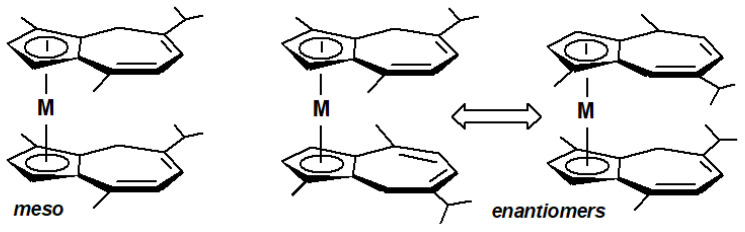
*Meso* and *racemic* isomers of bis(dihydroguaiazulenyl)metal complexes.

**Figure 8:**
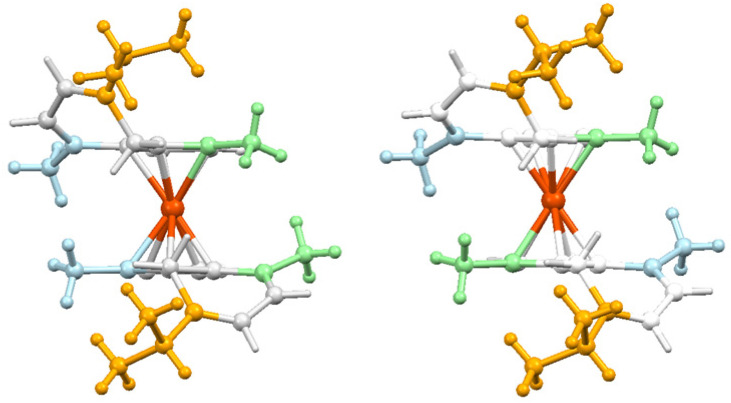
Molecular structures of (left) the *meso* and (right) a *racemic* isomer of bis(dihydroguaiazulenyl)iron.

The issue of chirality in these molecules becomes more apparent in the metallocene sandwich compounds shown in Figure 7, for which *meso* and *racemic* isomers have been isolated and fully characterised by X-ray crystallography (Figure 8). These include complexes possessing iron, ruthenium, cobalt or rhodium as the central metal.

**Scheme 72:**
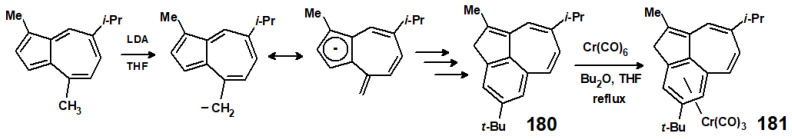
Synthesis of the (benz[*cd*]azulene)Cr(CO)_3_, **181**, derived from guaiazulene.

**Scheme 73:**
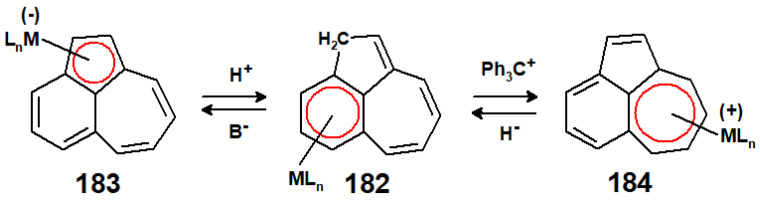
Possible haptotropic shifts over the benz[*cd*]azulene skeleton.

Unlike its parent azulene, guaiazulene is readily functionalised by the ready deprotonation of the 4-methyl group, whereby the negative charge is delocalised into the five-membered ring, thus generating a cyclopentadienide-type anion (Scheme 72). Treatment with 1-chloropinacolone, followed by Friedel–Crafts ring closure with AlCl_3_, yielded the benz[*cd*]azulene **180** that was characterised crystallographically as the η^6^-Cr(CO)_3_ complex, **181** [102]. This tricyclic system has the potential to exhibit η^6^ → η^5^ (**182** → **183**), or η^6^ → η^7^ (**182** → **184**) haptotropic migrations when deprotonated or treated with a hydride abstractor, such as the trityl cation (Scheme 73).

## 13. Metal Cluster Complexes of Terpenoids

### 13.1. Cobalt Cluster-Stabilised Carbocations

**Scheme 74:**
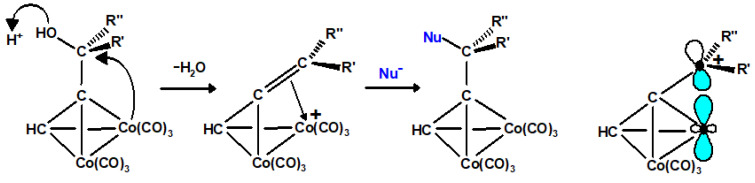
The Nicholas reaction, depicting overlap of a filled *d* orbital with the vacant *p* orbital on carbon.

The chemistry of metal clusters is voluminous, but we focus here on the syntheses and reactivity of bi- and tri-metallic derivatives of terpenoids, in particular, tetrahedral clusters derived from alkynes and metal carbonyl precursors. The Nicholas reaction [103] is based on the intermediacy of cobalt-stabilised propargyl cations that are susceptible to nucleophilic attack, thus allowing for a controlled enhancement of molecular complexity (Scheme 74).

**Scheme 75:**
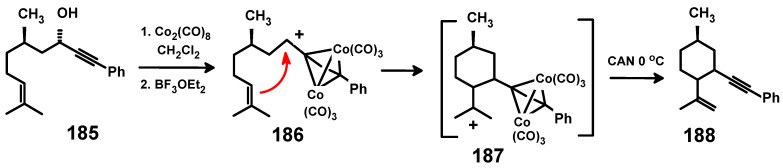
A metal cluster-promoted cyclisation reaction.

**Scheme 76:**
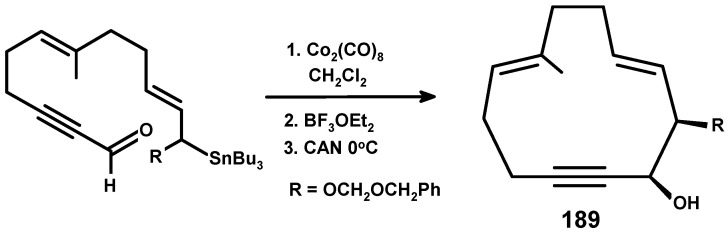
Cobalt-mediated cyclisation to form a cyclododecadienynol.

A typical example from the terpene field (Scheme 75) involves the addition of a zinc acetylide to citronellal in the presence of chiral ligand such as *N*-methylephredine, followed by the treatment of the alkyne **185** with dicobalt octacarbonyl to yield a tetrahedral alkyne-dicobalt cluster. Upon treatment with a Lewis acid, it forms the metal-stabilised propargyl cation **186**, that undergoes intramolecular cyclisation to **187**. Addition of ceric ammonium nitrate (CAN) leads to decomposition of the metal cluster and liberates the final product **188** [104]. Another fine example is Marshall’s synthesis of the cyclododecadienynol **189** (Scheme 76) [105].

### 13.2. X-ray Crystallographic Characterisation of Metal-Stabilised Bornyl Carbocations

**Scheme 77:**
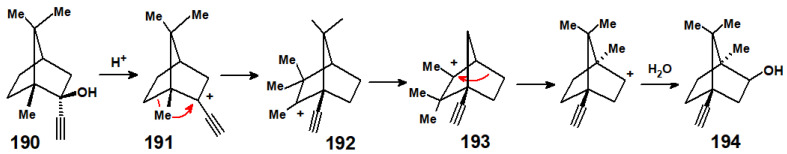
Sequential molecular rearrangements of 2-ethynylborneol.

Treatment of (1*R*)-(+)-camphor with an alkynyl anion yields the corresponding *endo*-2-alkynylborneol, whereby nucleophilic attack at the carbonyl proceeds so as to avoid steric interactions with the *gem*-dimethyl bridging moiety. As is well-known, complex molecular rearrangements are frequently encountered in terpene chemistry, and the protonation of *endo*-2-ethynylborneol **190** to form the corresponding propargyl cation, **191**, leads to a spectacular series of migrations that culminate with the alkynyl substituent which is no longer adjacent to the carbocationic site (Scheme 77). The initial Wagner–Meerwein (W-M) rearrangement, to form **192**, is followed by a 1,2-methyl migration (Nametkin shift) to **193**, and then a second W-M and hydrolysis to yield the final product, **194** [106].

**Scheme 78:**
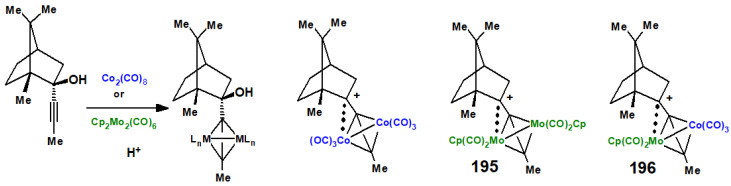
Characterisation of 2-bornyl cations preferentially stabilised by interaction with molybdenum.

When *endo*-2-propynylborneol was allowed to react with either dicobalt octacarbonyl or (cyclopentadienyl)dicarbonylmolybdenum dimer (Scheme 78), the resulting tetrahedral alkyne-dimetallic clusters isolated were each characterised by X-ray crystallography [107,108]. However, upon protonation, the metal-stabilised cationic clusters resisted skeletal rearrangement and were unambiguously identified spectroscopically. Moreover, the dimolybdenum cationic complex, **195**, was also characterised by X-ray crystallography, which revealed that the bornyl cation clearly leaned toward molybdenum such that the Mo-C(+) distance was only 2.74 Å. In the mixed Co-Mo cation, **196**, once again, the Mo-C(+) distance was short (2.91 Å), indicating that the molybdenum centre was the preferred site for cation stabilisation, a conclusion supported by calculations at the EHMO level [109]; this area has been reviewed by Gruselle [110].

### 13.3. Metal Cluster Complexes in the Fenchyl, Verbenyl, Menthyl and Geranyl Series

**Scheme 79:**
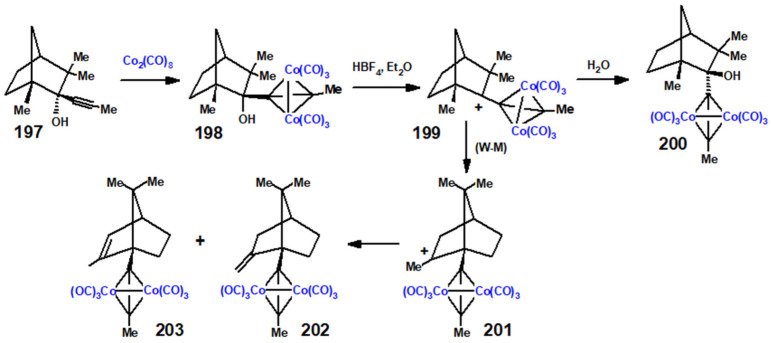
Rearrangement behaviour of the [(fenchyl)Co_2_(CO)_6_]^+^ cluster cation.

**Figure 9:**
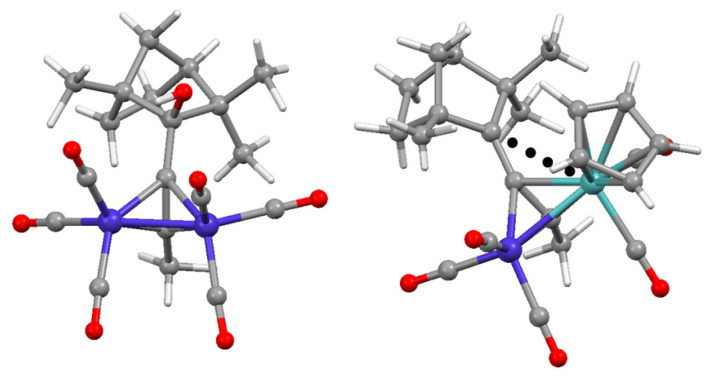
Structures of (**left**) the dicobalt-fenchyl cluster, **200**, and (**right**) the cobalt–molybdenum cation, **205**.

The situation becomes more interesting in the fenchyl case. Initial nucleophilic attack on fenchone by an alkynyl anion proceeds almost exclusively (95:5) to form the 2-*exo*-alkynol **197**. (Strictly speaking, we should perhaps designate this as the *endo* epimer since OH takes precedence over the C≡C linkage in the Cahn–Ingold–Prelog protocol.) Treatment of **197** with Co_2_(CO)_8_ yielded the expected complex, **198**, that was relatively unstable until protonated to form the cationic complex **199**; however, when rapidly quenched with water, the product was characterised by X-ray crystallography as **200** (Figure 9), the epimer of the original alkynol (Scheme 79). This is presumably attributable to the steric strain engendered between the bulky cluster fragment and the *gem*-dimethyl group at C(3). However, when the cation **199** was allowed to stand for an extended time, it underwent a W-M shift to form **201**, thereby placing the cluster fragment at C(1) with the carbocationic site at C(6), prior to elimination, leading to the alkenes **202** and **203** [111].

**Scheme 80:**
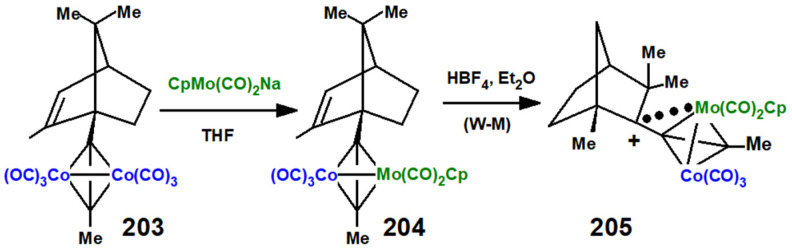
Rearrangement behaviour of the [(fenchyl)(C_5_H_5_)MoCo(CO)_5_]^+^ cluster cation.

**Figure 10:**
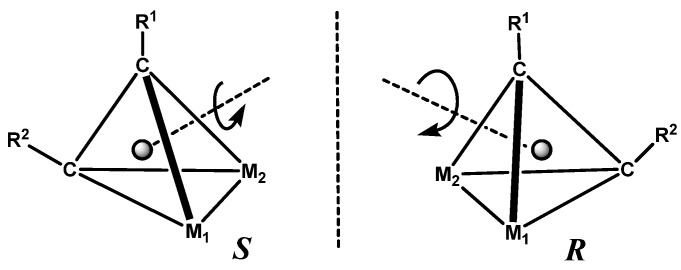
Designation of the absolute configuration of a tetrahedral organometallic cluster.

Now, displacement of a tricarbonylcobalt vertex in **203** by a (cyclopentadienyl)dicarbonyl molybdenum moiety to form the mixed metal cluster **204**, followed by reprotonation, brought about a retro W-M migration to yield **205**, thus placing the molybdenum adjacent to the carbocation at C(2) as in Scheme 80. The structure was verified crystallographically (Figure 9) and revealed that the Mo-C(+) interaction was now directed to the *exo* face of the fenchyl framework, contrary to the previously observed *endo* interactions seen in the cationic bornyl clusters. The absolute configuration of the chiral cluster moiety in **205** can be designated as *R* relative to a dummy atom positioned within the tetrahedron (Figure 10) [112]; CIP rules give the order of precedence as Mo > Co > C-fenchyl > C-methyl. Evidently, the differing abilities of the molybdenum and cobalt vertices to alleviate the positive charge on carbon and stabilise the structure ultimately control the direction of the Wagner–Meerwein rearrangements [111].

**Figure 11:**
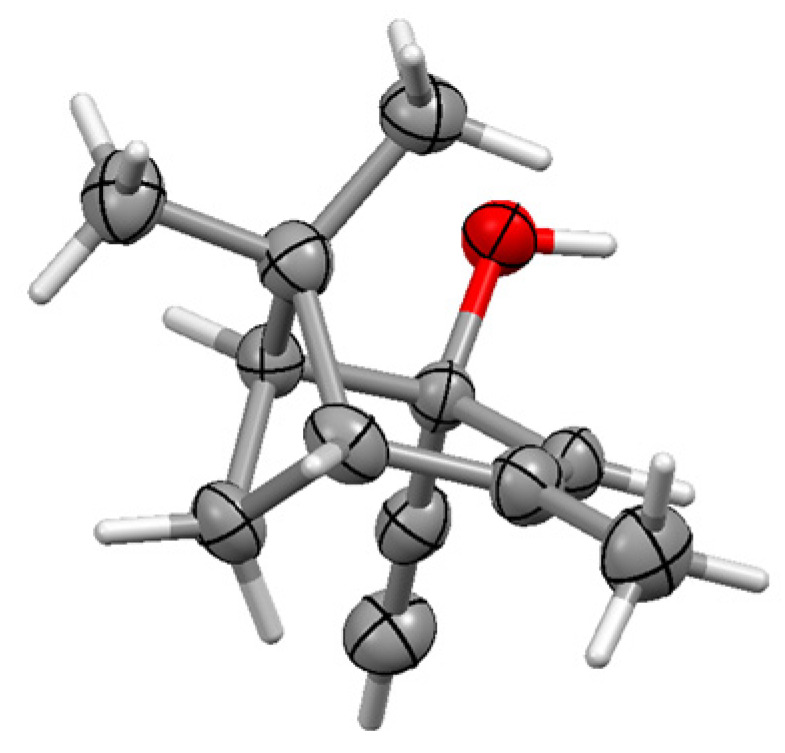
Molecular structure of 2-ethynyl-verbenol, **206**.

**Scheme 81:**
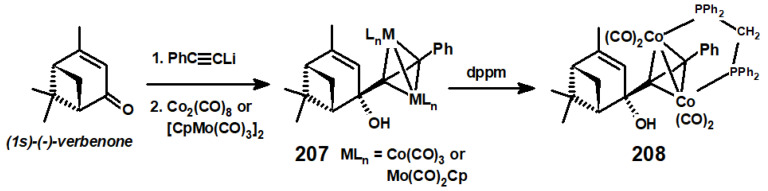
Reactions of verbenone with alkynyl anions leading to tetrahedral bimetallic clusters.

In related work on other chiral monoterpenoids, we should mention verbenone that possesses a pinene skeleton and is found in a variety of plants, especially rosemary. It has a pleasant odour and is widely used in perfumery, aromatherapy, herbal teas and spices and herbal remedies. The ketone itself plays an important role in the control of the southern Bark Beetle, and *cis* and *trans* verbenols are pheromones for *Ips paraconfusus*, another species of bark beetle. Alkynyl anion attack on verbenone occurs exclusively on the face opposite to the *gem*-dimethyl bridge to form the alkynol, **206**, shown in Figure 11 [113]. The addition of Co_2_(CO)_8_ or [CpMo(CO)_3_]_2_ delivers the alkyne-dimetallic tetrahedral clusters, **207**, and further reaction in the former with bis(diphenylphosphino)methane (dppm) yields the complex **208** that exhibits two ^31^P NMR signals arising from the diastereotopic character of the phosphorus nuclei (Scheme 81).

**Scheme 82:**
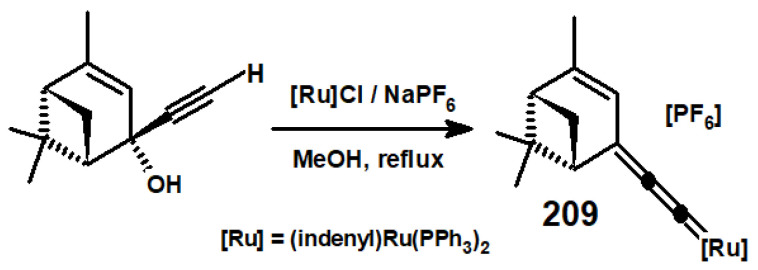
Formation of the allenylidene–ruthenium complex, **209**, from 2-ethynyl-verbenol.

2-Ethynyl-verbenol also reacts with the indenyl reagent, (η^5^-C_9_H_7_)(PPh_3_)_2_RuCl, leading ultimately to the allenylidene cationic complex, **209** (Scheme 82), that is also susceptible to nucleophilic attack [114], somewhat analogous to the reactions of cobalt-stabilised carbocations.

**Scheme 83:**
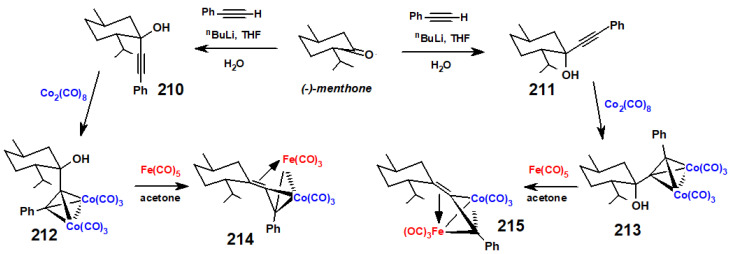
Alkynyl-menthol dicobalt and mixed iron–cobalt tetrahedral cluster complexes.

In contrast to the earlier cases, alkynyl anion attack on menthone yields a mixture of alkynyl-menthols, **210** and **211**, in which the predominant isomer is the latter, whereby the alkynyl substituent occupies the equatorial site. The original attribution of these isomers was based on NMR data of their derived allenyl-phosphine oxides [115], and the X-ray crystallographic structure determinations of their phenylethynyl-Co_2_(CO)_6_ tetrahedral clusters, **212** and **213**, respectively, validated these assignments [116]. As anticipated, protonation of these alkynol clusters yielded cationic complexes but, since they were not amenable to X-ray structural analysis, they were also treated with iron pentacarbonyl in acetone, a reaction previously shown to yield neutral mixed-metal Fe-Co complexes [117] in which Fe(CO)_3_ replaced the isoelectronic, and isolobal, [Co(CO)_3_]^+^ fragment. As depicted in Scheme 83, the structures of the neutral Fe-Co clusters, **214** and **215** (established X-ray crystallographically), provide excellent models for the cobalt-stabilised menthyl carbocations analogous to those found in their bornyl and fenchyl counterparts.

**Scheme 84:**
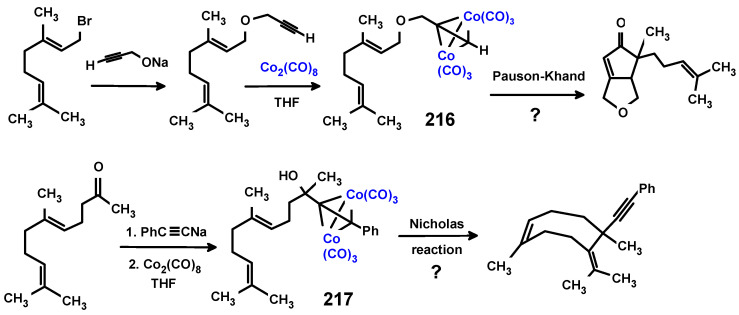
Dicobalt hexacarbonyl complexes from alkynyl derivatives of geraniol.

Finally, we note the preparation of tetrahedral clusters **216** and **217**, arising from the reaction of Co_2_(CO)_8_ with propargyl geranyl ether, or the alkynol derived from geranyl acetone; these complexes may have potential for Pauson–Khand cyclisation, or a Nicholas reaction (Scheme 84).

**Scheme 85:**
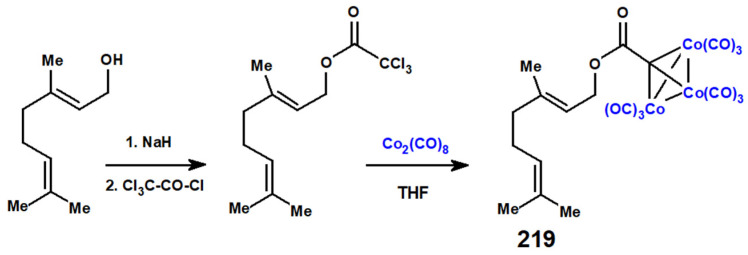
Synthesis of a geranyl-carbynyltricobalt-nonacarbonyl cluster, **219**.

**Scheme 86:**
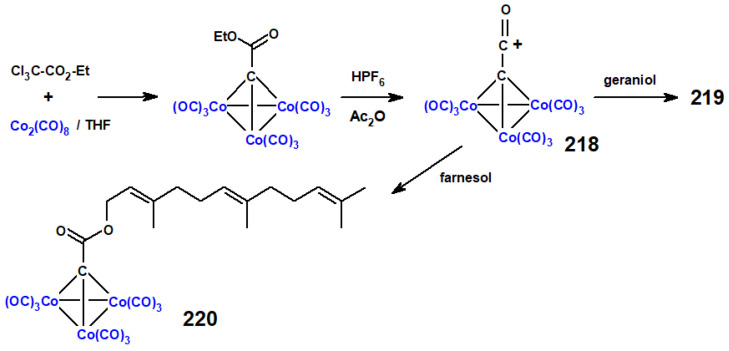
Synthesis of geranyl- and farnesyl-carbynyltricobalt-nonacarbonyl clusters.

The syntheses of carbynyl-tricobalt-nonacarbonyl clusters, RCCo_3_(CO)_9_, are readily achieved either by direct reaction of Co_2_(CO)_8_ with the appropriate trichloromethyl precursor (Scheme 85), or by nucleophilic attack on the acylium cation, **218**, derived from an ester (Scheme 86). Both of these approaches have been used, thereby leading successfully to tricobalt-nonacarbonyl clusters bearing geranyl, **219**, or farnesyl, **220**, substituents [118].

## 14. Concluding Remarks

The evolution of the organometallic chemistry of terpenes and terpenoids has been dramatic, from the early (1960s and 1970s) syntheses of mono- and sesqui-terpenes which involved taking advantage of the newly developed carbon–carbon coupling procedures using nickel or iron carbonyls, or a variety of palladium-based reagents, to the more recently reported efficient routes to industrially or medicinally important molecules. These studies have been complemented by the elucidation of mechanistic details, as well as the discovery of unexpected molecular rearrangements brought about by reactions of terpenes with a wide variety of organometallic species.

The initial burst of publications in this area, with 37 in each period of decades, 1966–1975 and 1976–1985, was followed by a period of retrenchment with 21 reports from 1986–2005, and 28 more since then. However, looking to the future and hoping for enlightenment, we have tried to focus on very recent publications that may stimulate further breakthroughs in the area. These include new industrial applications, enhanced methods of structural determination and the re-evaluation of earlier work by using today’s spectroscopic techniques.

In many countries, access to the vitamins A, E and K, as well as important materials such as geraniol, linalool, farnesol and phytol, is limited, owing to the lack of industrially competitive technologies for their syntheses, thus necessitating reliance on imports. The development of simple and efficient catalytic methods for the production of dimers, trimers and tetramers of isoprene as routes to regular acyclic *trans*-isoprenoids is a growing area, and is the focus of a 2023 review [119].

**Scheme 87:**
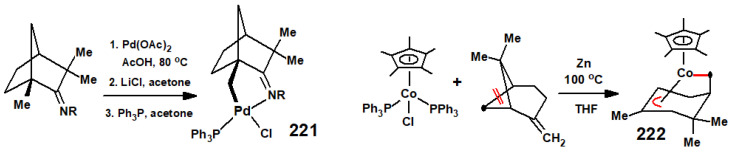
Complexes possessing a direct terpenoid metal σ-bond.

As noted in the introduction, chiral terpenoids have long been incorporated into coordination compounds as part of the search for improved catalysts in asymmetric syntheses, and these have since been modified to possess a metal carbon σ-bond, thereby linking the metal directly to the asymmetric moiety. Typically, readily available and inexpensive (1*R*)-(-)-fenchone is convertible into an imine that, when cyclopalladated, yields systems such as **221** that can easily be further elaborated (Scheme 87) [120]. A different application of this approach involves the reaction of (C_5_Me_5_)Co(PPh_3_)_2_ with β-pinene, whereupon bond fission leads to **222** containing both η^1^- and η^3^-allyl attachments to cobalt, a structure closely analogous to that of the iron complex **161** shown in Figure 4. However, the goal here is very different; this molecule, and a number of its variants, have been found to be ideal for the low-temperature atomic-layer deposition of cobalt-containing thin films to cover complex 3D structures [121].

**Figure 12:**
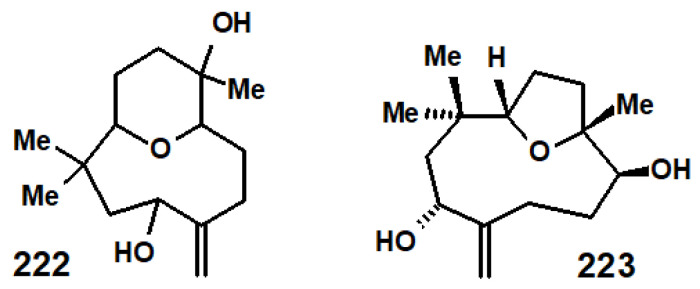
Proposed, **222**, and revised structure, **223**, of *nordine*.

One should always respect and admire those pioneers in the chemistry of terpenoids who attempted to determine the structures of complex organic and organometallic molecules without access to the spectroscopic equipment now available. Much of the earlier work is based on ^1^H NMR data at 60 or 90 MHz, whereas nowadays, access to multi-nuclear, multi-pulse FT instruments operating at 600 MHz and above is routine. In a typical example (Figure 12), the proposed structure, **222**, of the sesquiterpene *nordine*, isolated from the bark or roots of the evergreen shrub *Anaxagorea javanica* Blume, has since been reassigned as **223**, based on the full range of two-dimensional NMR techniques, and then confirmed by X-ray crystallography [122]. We can anticipate that the reinvestigation of contentious structures by taking advantage of the latest analytical techniques will be an ongoing activity.

Perhaps the greatest advances in structural determination techniques have taken place in X-ray crystallography. Of course, there have been enormous technological improvements, such as more powerful rotating anode X-ray sources, more sensitive charge-coupled or area detectors, infinitely better computational facilities, and modern direct method programs, but many common mono- or sesqui-terpenes are low-melting and volatile, characteristics that enhance their role in perfumery. The currently available structures of such materials are frequently based on crystallizable derivatives, such as the humulene complex with silver nitrate [123], or as its di-epoxide (Figure 5) [93]. This difficulty has been largely overcome recently by the application of the crystalline sponge method, whereby the compound of interest is included as a guest into a porous metal organic framework consisting of, for example, ZnI_2_ and 2,4,6-tri(pyridin-4-yl)-1,3,5-triazine.

**Figure 13:**
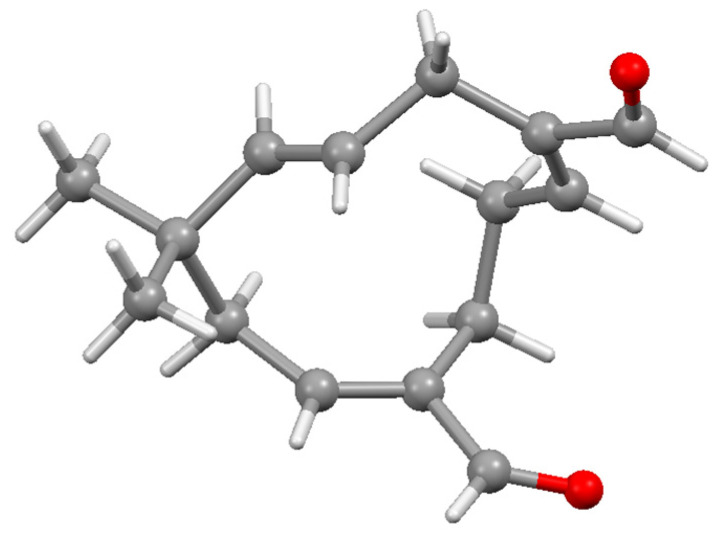
Molecular structure of humulene-2,9-di-aldehyde.

**Figure 14:**
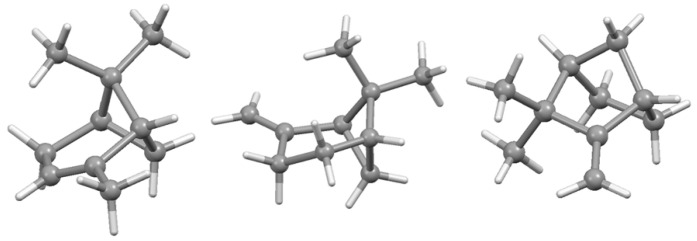
Molecular structures of (left) α-pinene, (centre) β-pinene, and (right) camphene, acquired using the crystalline sponge X-ray crystallographic method.

An impressive demonstration of the power of this approach is the report by Makoto Fujita at the University of Tokyo, the originator of the crystalline sponge method, describing the identification of a multitude of products arising from the oxidation of α-humulene that yields a number of epoxides, and also aldehydes derived from the single methyls, all of which were structurally characterised [124]. A typical example is shown in Figure 13. Other recently reported structures of low-melting monoterpenes include α- and β-pinene, and camphene, as shown in Figure 14 [125].

**Figure 15:**
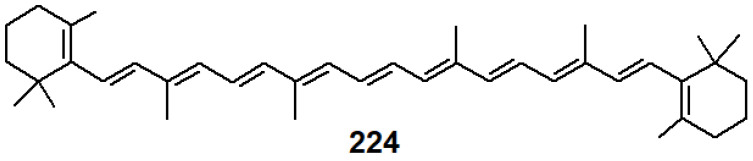
Structure of β-carotene, **224**.

**Figure 16:**
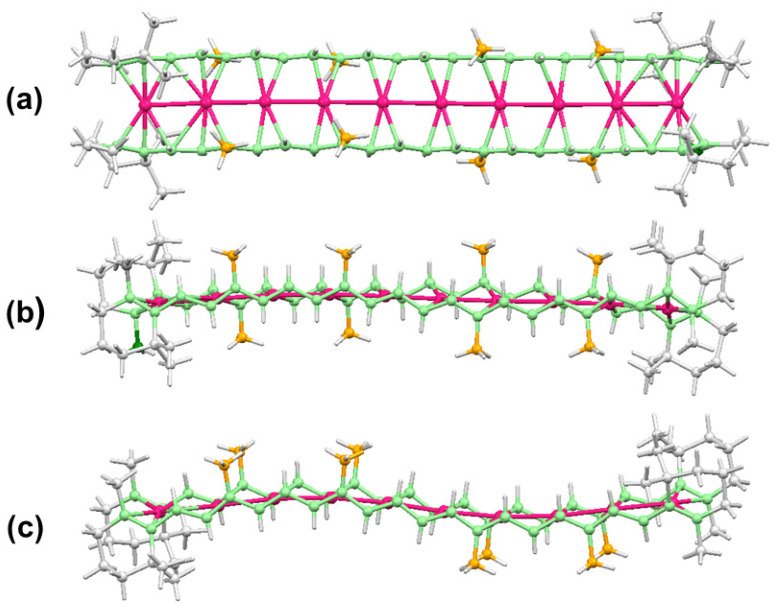
X-ray crystal structures of the carotene-Pd_10_ chain complexes: (**a**) *rac*-side view, (**b**) *rac*-top view, (**c**) *meso*-top view.

In closing, we feature the spectacular report from a Japan–Spain collaboration of the multinuclear binding ability of β-carotene, **224** (Figure 15), to capture as many as ten palladium atoms in a metal-bonded chain sandwich [126]. This exists in both *meso* (*C*_2h_) and *racemic* (*D*_2_) forms, and both have been fully characterised spectroscopically and by X-ray crystallography (Figure 16). Even more impressively, the system can be selectively de-metallated under a CO atmosphere, and palladium atoms can then be replaced by platinum atoms—a quite brilliant achievement!
